# Supplementary material for: The GARP complex is required for cellular sphingolipid homeostasis
Source: eLife. 2015 Sep 10;4:e08712. doi: 10.7554/eLife.08712 (PMC4600884; doi:10.7554/eLife.08712)
Supplement: Supplementary file 1. — List of all yeast strains used in this study. DOI: http://dx.doi.org/10.7554/eLife.08712.018 [file elife08712s001.docx]

| Strain | Genotype | Reference |
| --- | --- | --- |
| TWY70 | *MATa his3Δ 1 leu2Δ 0 lys2Δ 0 ura3Δ 0* | Walther et al., 2006 |
|  | *MATa his3Δ 1 leu2Δ 0 lys2Δ 0 ura3Δ 0 vps51Δ::KAN®* | Winzler et al., 1999 |
|  | *MATa his3Δ 1 leu2Δ 0 lys2Δ 0 ura3Δ 0 vps52Δ::KAN®* | Winzler et al., 1999 |
|  | *MATa his3Δ 1 leu2Δ 0 lys2Δ 0 ura3Δ 0 vps53Δ::KAN®* | Winzler et al., 1999 |
|  | *MATa his3Δ 1 leu2Δ 0 lys2Δ 0 ura3Δ 0 vps54Δ::KAN®* | Winzler et al., 1999 |
| TWY3058 | *MATα his3Δ 1 leu2Δ 0 lys2Δ 0 ura3Δ 0 vps52Δ::NAT®* | this study |
| TWY3386 | *MATa his3Δ 1 leu2Δ 0 met15Δ 0 ura3Δ 0 erg3D::KAN®* | this study |
| TWY3442 | *MATa his3Δ 1 leu2Δ 0 lys2Δ 0 ura3Δ 0 ORM1-HA::HIS* | this study |
| TWY3495 | *MATa his3Δ 1 leu2Δ 0 lys2Δ 0 ura3Δ vps53Δ::NAT®* | this study |
| TWY3564 | *MATa his3Δ 1 leu2Δ 0 lys2Δ 0 ura3Δ 0 Orm1-HA::NAT vps53Δ::HPH* | this study |
| TWY3625 | *MATa his3Δ 1 leu2Δ 0 lys2Δ 0 ura3Δ 0 VPS10-GFP::KAN® SEC7-tomato::HIS vps53Δ::NAT® pRS416* | this study |
| TWY3626 | *MATa his3Δ 1 leu2Δ 0 lys2Δ 0 ura3Δ 0 VPS10-GFP::KAN® SEC7-tomato::HIS vps53Δ::NAT® pRS416_VPS53* | this study |
| TWY3627 | *MATa his3Δ 1 leu2Δ 0 lys2Δ 0 ura3Δ 0 VPS10-GFP::KAN® SEC7-tomato::HIS vps53Δ::NAT® pRS416_vps53(Q624R)* | this study |
| TWY3628 | *MATa his3Δ 1 leu2Δ 0 lys2Δ 0 ura3Δ 0 VPS10-GFP::KAN® VPS17-tomato::HIS vps53Δ::NAT® pRS416* | this study |
| TWY3629 | *MATa his3Δ 1 leu2Δ 0 lys2Δ 0 ura3Δ 0 VPS10-GFP::KAN® VPS17-tomato::HIS vps53Δ::NAT® pRS416_VPS53* | this study |
| TWY3630 | *MATa his3Δ 1 leu2Δ 0 lys2Δ 0 ura3Δ 0 VPS10-GFP::KAN® VPS17-tomato::HIS vps53Δ::NAT® pRS416_vps53(Q624R)* | this study |
| TWY3631 | *MATa ura3-52; trp1Δ 2; leu2-3,112; his3-11; ade2-1; can1-102 VMA1-mars::NAT® VPS10-GFP::HIS vps53Δ::HPH® pRS416* | this study |
| TWY3632 | *MATa ura3-52; trp1Δ 2; leu2-3,112; his3-11; ade2-1; can1-102 VMA1-mars::NAT® VPS10-GFP::HIS vps53Δ::HPH® pRS416_VPS53* | this study |
| TWY3633 | *MATa ura3-52; trp1Δ 2; leu2-3,112; his3-11; ade2-1; can1-102 VMA1-mars::NAT® VPS10-GFP::HIS vps53Δ::HPH® pRS416_vps53(Q624R)* | this study |
| TWY3695 | *MATa his3Δ 1 leu2Δ 0 met15Δ 0 ura3Δ pRS416_VPS53_Q624R::URA* | this study |
| TWY3696 | *MATa his3Δ 1 leu2Δ 0 met15Δ 0 ura3Δ galS_GFP_YPC1::NAT®* | this study |
| TWY3697 | *MATa his3Δ 1 leu2Δ 0 met15Δ 0 ura3Δ pRS426::URA* | this study |
| TWY3698 | *MATa his3Δ 1 leu2Δ 0 met15Δ 0 ura3Δ vps53Δ::NAT® pRS426::URA* | this study |
| TWY3699 | *MATa his3Δ 1 leu2Δ 0 met15Δ 0 ura3Δ pRS426_galS_GFP_YPC1::URA* | this study |
| TWY3700 | *MATa his3Δ 1 leu2Δ 0 met15Δ 0 ura3Δ vps53Δ::NAT® pRS426_galS_GFP_YPC1::URA* | this study |

**Supplementary Table 1: List of all yeast strains used in this study**
