# Supplementary material for: The GARP complex is required for cellular sphingolipid homeostasis
Source: eLife. 2015 Sep 10;4:e08712. doi: 10.7554/eLife.08712 (PMC4600884; doi:10.7554/eLife.08712)
Supplement: Supplementary file 2. — List of all plasmids used in this study. DOI: http://dx.doi.org/10.7554/eLife.08712.019 [file elife08712s002.docx]

|  | |  |
| --- | --- | --- |
|  |  |  |
| Number | Plasmid | Reference |
| TWP103 | pRS426 | Sikorski and Hieter,1989 |
| TWP355 | pRS416 | Sikorski and Hieter,1989 |
| TWP761 | pRS416_VPS53 | this study |
| TWP762 | pRS416_VPS53_S790A | this study |
| TWP763 | pRS416_VPS53_S790D | this study |
| TWP764 | pRS416_VPS53_Q624R | this study |
| TWP765 | pRS426_galS_GFP_YPC1 | this study |

**Supplementary Table 2: List of all plasmids used in this study**
