# Supplementary material for: The GARP complex is required for cellular sphingolipid homeostasis
Source: eLife. 2015 Sep 10;4:e08712. doi: 10.7554/eLife.08712 (PMC4600884; doi:10.7554/eLife.08712)
Supplement: Supplementary file 3. — List of all hits identified in the chemical genomic screen. DOI: http://dx.doi.org/10.7554/eLife.08712.020 [file elife08712s003.docx]

| **Supressors** | | | | | | |
| --- | --- | --- | --- | --- | --- | --- |
| **ORF** | **Gene name** | **Mutation** | **WT.mean** | **MYR.mean** | **T-score** | **Significance** |
| YDR027C | VPS54 | DELETION | 634.811514 | 1749.878378 | 46.84089045 | 1.12E-23 |
| YDL192W | ARF1 | DELETION | 728.2255968 | 1524.467711 | 33.44794008 | 8.30E-13 |
| YML065W | ORC1 | DAMP-D3205 | 830.6214462 | 1597.710286 | 32.22329127 | 5.45E-12 |
| YDR484W | VPS52 | DELETION-31620 | 490.4203385 | 1211.996363 | 30.31142314 | 8.99E-11 |
| YDL015C | TSC13 | DAMP-D2108 | 621.5447153 | 1330.717144 | 29.79038221 | 1.88E-10 |
| YDR484W | VPS52 | DELETION-41519 | 520.2803888 | 1228.88463 | 29.7665142 | 1.94E-10 |
| YGR264C | MES1 | DAMP-D1103 | 776.979686 | 1481.421383 | 29.59165717 | 2.48E-10 |
| YNR038W | DBP6 | DAMP | 715.3267222 | 1406.73317 | 29.04408222 | 5.27E-10 |
| YJL029C | VPS53 | DELETION | 558.0214098 | 1236.770484 | 28.5123808 | 1.08E-09 |
| YDL015C | TSC13 | DAMP-D2107 | 643.0631655 | 1310.204357 | 28.0247656 | 2.07E-09 |
| YOR272W | YTM1 | DAMP-D3026 | 1091.705198 | 1757.702121 | 27.97669803 | 2.21E-09 |
| YDL217C | TIM22 | DAMP | 706.6636377 | 1365.793872 | 27.68824732 | 3.22E-09 |
| YDR126W | SWF1 | DELETION | 858.5011003 | 1514.860543 | 27.57185397 | 3.75E-09 |
| YLR002C | NOC3 | DAMP | 875.420581 | 1517.915476 | 26.98944248 | 7.93E-09 |
| YBR087W | RFC5 | DAMP-D0912 | 674.6417732 | 1300.398965 | 26.28633766 | 1.92E-08 |
| YMR113W | FOL3 | DAMP | 620.6841225 | 1242.88446 | 26.13692396 | 2.31E-08 |
| YLR305C | STT4 | DAMP-D2413 | 671.15424 | 1270.464537 | 25.17537636 | 7.42E-08 |
| YDR381W | YRA1 | DAMP-D1146 | 567.1612776 | 1166.045374 | 25.15747283 | 7.58E-08 |
| YNR047W | FPK1 | DELETION | 738.3765133 | 1322.64601 | 24.54355375 | 1.56E-07 |
| YCR035C | RRP43 | DAMP | 659.888545 | 1228.102253 | 23.86909423 | 3.39E-07 |
| YGR013W | SNU71 | DAMP-D0332 | 694.1287634 | 1234.239209 | 22.68855354 | 1.25E-06 |
| YJL097W | PHS1 | DAMP-D2708 | 801.027047 | 1339.231794 | 22.60850038 | 1.37E-06 |
| YDL083C | RPS16B | DELETION-43137 | 886.3074941 | 1410.528599 | 22.02108603 | 2.55E-06 |
| YDR381W | YRA1 | DAMP-D1145 | 493.961169 | 1013.281447 | 21.81521578 | 3.17E-06 |
| YGL003C | CDH1 | DELETION | 840.7531352 | 1354.158969 | 21.56676625 | 4.10E-06 |
| YGR013W | SNU71 | DAMP-D0331 | 703.4837796 | 1213.001976 | 21.40345733 | 4.85E-06 |
| YGR264C | MES1 | DAMP-D1104 | 852.6558495 | 1353.059344 | 21.02057379 | 7.16E-06 |
| YKL139W | CTK1 | DELETION | 591.5511107 | 1082.799281 | 20.63598378 | 1.05E-05 |
| YBL014C | RRN6 | DAMP-D0127 | 839.3491758 | 1322.144654 | 20.28090947 | 1.49E-05 |
| YLR056W | ERG3 | DELETION | 568.0530706 | 1043.243447 | 19.96144005 | 2.03E-05 |
| YJR072C | NPA3 | DAMP-D3108 | 737.7073222 | 1205.872427 | 19.66632775 | 2.69E-05 |
| YJL167W | ERG20 | DAMP-D2715 | 850.1111473 | 1317.175669 | 19.62009528 | 2.81E-05 |
| YMR268C | PRP24 | DAMP-D0627 | 426.6987223 | 887.8874452 | 19.37326913 | 3.54E-05 |
| YOR081C | TGL5 | DELETION | 833.4452629 | 1288.847767 | 19.13020599 | 4.44E-05 |
| YGL001C | ERG26 | DAMP-D2745 | 755.8249004 | 1208.964628 | 19.035153 | 4.85E-05 |
| YBR160W | CDC28 | DAMP-D1308 | 681.3880054 | 1353.250211 | 19.02648034 | 4.89E-05 |
| YDL118W | YDL118W | DELETION | 739.3445307 | 1180.912223 | 18.54904363 | 7.54E-05 |
| YNR035C | ARC35 | DAMP-D2126 | 899.5588242 | 1337.369303 | 18.39121343 | 8.69E-05 |
| YJR072C | NPA3 | DAMP-D3107 | 792.3670004 | 1218.726259 | 17.91017919 | 0.000132688 |
| YJL034W | KAR2 | DAMP-D2320 | 839.0293355 | 1264.775156 | 17.88441033 | 0.000135696 |
| YJL050W | MTR4 | DAMP-D0941 | 648.0052403 | 1073.361243 | 17.86803516 | 0.000137641 |
| YMR220W | ERG8 | DAMP-D0603 | 988.1989115 | 1410.524299 | 17.74072733 | 0.00015368 |
| YOL026C | MIM1 | DAMP | 488.2836844 | 910.6070899 | 17.7406441 | 0.000153691 |
| YBL093C | ROX3 | DELETION | 1031.226724 | 1453.310346 | 17.73057144 | 0.000155032 |
| YJL050W | MTR4 | DAMP-D0942 | 645.9802761 | 1066.256014 | 17.65462723 | 0.000165507 |
| YGL171W | ROK1 | DAMP-D0312 | 1104.732352 | 1523.195299 | 17.57847684 | 0.000176676 |
| YPL106C | SSE1 | DELETION | 815.1830488 | 1233.071703 | 17.55435242 | 0.00018036 |
| YJL051W | IRC8 | DELETION | 1062.265673 | 1475.329128 | 17.35165908 | 0.000214291 |
| YCR034W | FEN1 | DELETION | 661.7486329 | 1289.980351 | 17.16387653 | 0.000250995 |
| YNL111C | CYB5 | DELETION-41727 | 907.710896 | 1313.803302 | 17.05882446 | 0.000274021 |
| YJL034W | KAR2 | DAMP-D2319 | 862.3652349 | 1262.373848 | 16.80326109 | 0.00033857 |
| YFR001W | LOC1 | DELETION | 404.9023499 | 802.885814 | 16.71819014 | 0.000363039 |
| YBR087W | RFC5 | DAMP-D0911 | 690.8969119 | 1345.095455 | 16.61898815 | 0.000393659 |
| YNL135C | FPR1 | DELETION | 722.8702909 | 1116.800307 | 16.54791593 | 0.000417061 |
| YML102W | CAC2 | DELETION | 777.3274057 | 1169.840812 | 16.48840804 | 0.000437649 |
| YMR117C | SPC24 | DAMP-D0407 | 781.7008485 | 1172.95841 | 16.43565348 | 0.00045669 |
| YIL019W | FAF1 | DAMP-D1512 | 856.4565214 | 1247.594847 | 16.43064469 | 0.000458538 |
| YDR335W | MSN5 | DELETION | 774.9624243 | 1166.090651 | 16.43022048 | 0.000458694 |
| YDL148C | NOP14 | DAMP-D2511 | 683.9391569 | 1261.876021 | 16.41147603 | 0.000465672 |
| YHR012W | VPS29 | DELETION | 937.3694501 | 1327.559867 | 16.39082568 | 0.000473475 |
| YBR160W | CDC28 | DAMP-D1307 | 693.0361555 | 1389.094061 | 16.34282475 | 0.000492084 |
| YOR068C | VAM10 | DELETION | 714.4883083 | 1101.960198 | 16.27662784 | 0.000518869 |
| YGR099W | TEL2 | DAMP-D0724 | 824.6744301 | 1210.030678 | 16.18775555 | 0.000556968 |
| YFR028C | CDC14 | DAMP | 692.1313158 | 1077.368141 | 16.18273892 | 0.000559195 |
| YDR093W | DNF2 | DELETION | 893.6568684 | 1276.858625 | 16.09725127 | 0.000598433 |
| YMR103C | YMR103C | DELETION | 935.4317501 | 1316.682794 | 16.01530723 | 0.000638437 |
| YPR131C | NAT3 | DELETION | 525.4706436 | 904.9334964 | 15.94019022 | 0.000677279 |
| YBR191W | RPL21A | DELETION-30638 | 541.0401595 | 918.129452 | 15.84048349 | 0.000732235 |
| YLL033W | IRC19 | DELETION | 816.4566468 | 1192.958749 | 15.81581722 | 0.000746455 |
| YEL033W | MTC7 | DELETION | 725.0446043 | 1101.361847 | 15.80805181 | 0.000750985 |
| YLR005W | SSL1 | DAMP-D1229 | 630.7340985 | 1336.915477 | 15.77119858 | 0.000772832 |
| YJL053W | PEP8 | DELETION | 905.0087011 | 1280.121068 | 15.75743828 | 0.000781139 |
| YDL136W | RPL35B | DELETION | 639.9718769 | 1013.542282 | 15.69266473 | 0.000821369 |
| YHL031C | GOS1 | DELETION | 727.5033802 | 1099.993436 | 15.64728221 | 0.000850692 |
| YCR033W | SNT1 | DELETION-30226 | 765.4726732 | 1136.652203 | 15.59223063 | 0.000887565 |
| YDL033C | SLM3 | DELETION-32022 | 797.5423164 | 1168.443058 | 15.58051948 | 0.000895598 |
| YNR035C | ARC35 | DAMP-D2125 | 844.5678236 | 1213.05875 | 15.47928979 | 0.000967894 |
| YLR339C | YLR339C | DAMP | 874.2632341 | 1241.113738 | 15.41038016 | 0.001020159 |
| YLR048W | RPS0B | DELETION | 564.4436015 | 929.2363557 | 15.32393975 | 0.001089414 |
| YER166W | DNF1 | DELETION-43226 | 1370.068507 | 1974.450857 | 15.29738336 | 0.001111548 |
| YIL019W | FAF1 | DAMP-D1511 | 834.0215652 | 1197.729084 | 15.27835198 | 0.001127666 |
| YPL120W | VPS30 | DELETION | 744.3203484 | 1108.014412 | 15.27778678 | 0.001128148 |
| YBR286W | APE3 | DELETION | 842.3468406 | 1203.157585 | 15.15666646 | 0.001235933 |
| YLR130C | ZRT2 | DELETION | 714.6115877 | 1075.142161 | 15.14489726 | 0.001246898 |
| YDR302W | GPI11 | DAMP-D1109 | 773.2341761 | 1130.9109 | 15.02501489 | 0.001363822 |
| YML024W | RPS17A | DELETION | 478.9504908 | 836.3696211 | 15.0141941 | 0.001374859 |
| YLR061W | RPL22A | DELETION | 479.625632 | 836.9406066 | 15.00981881 | 0.001379345 |
| YDR407C | TRS120 | DAMP-D1510 | 750.2540369 | 1310.48089 | 14.85629472 | 0.001545564 |
| YOL018C | TLG2 | DELETION | 558.5600844 | 1304.200594 | 14.65545257 | 0.001790879 |
| YDR390C | UBA2 | DAMP | 570.8612061 | 915.9190462 | 14.49493032 | 0.002012149 |
| YKR092C | SRP40 | DELETION | 704.052153 | 1048.519017 | 14.47010502 | 0.002048527 |
| YHR197W | RIX1 | DAMP-D1508 | 752.4343937 | 1096.423376 | 14.45003055 | 0.002078383 |
| YMR202W | ERG2 | DELETION-42431 | 685.3164058 | 1028.568253 | 14.41906552 | 0.002125221 |
| YJL011C | RPC17 | DAMP-D2304 | 686.4436848 | 1028.953175 | 14.38788115 | 0.002173366 |
| YOR042W | CUE5 | DELETION | 781.8255648 | 1123.685644 | 14.3606012 | 0.002216302 |
| YOR136W | IDH2 | DELETION | 437.1661783 | 777.4938819 | 14.2962303 | 0.002320712 |
| YJR091C | JSN1 | DELETION | 911.4940396 | 1251.771102 | 14.294103 | 0.002324238 |
| YDR407C | TRS120 | DAMP-D1509 | 765.4963062 | 1293.667462 | 14.23438142 | 0.002425257 |
| YLR055C | SPT8 | DELETION | 685.4004464 | 1022.963821 | 14.18010843 | 0.002520528 |
| YDL195W | SEC31 | DAMP-D2527 | 808.49599 | 1145.722819 | 14.1659711 | 0.0025459 |
| YLL035W | GRC3 | DAMP | 707.1913391 | 1044.210766 | 14.1572587 | 0.002561653 |
| YPR101W | SNT309 | DELETION | 831.6624419 | 1168.464601 | 14.14813187 | 0.00257825 |
| YNL323W | LEM3 | DELETION | 720.9440146 | 1795.352176 | 14.11107346 | 0.002646656 |
| YJL129C | TRK1 | DELETION-31231 | 377.3492214 | 712.6531388 | 14.08519487 | 0.002695405 |
| YDR028C | REG1 | DELETION | 721.5457666 | 1313.298794 | 14.06601402 | 0.002732065 |
| YDL033C | SLM3 | DELETION-41921 | 868.1944825 | 1202.335533 | 14.03634602 | 0.002789667 |
| YHR197W | RIX1 | DAMP-D1507 | 754.1611071 | 1087.77293 | 14.01411462 | 0.002833554 |
| YJL097W | PHS1 | DAMP-D2707 | 799.125106 | 1325.518883 | 13.95289596 | 0.002957686 |
| YBR266C | SLM6 | DELETION | 443.5704978 | 772.9631694 | 13.8368797 | 0.003206633 |
| YNL067W | RPL9B | DELETION | 757.0085296 | 1082.193106 | 13.6601092 | 0.00362281 |
| YHL002W | HSE1 | DELETION | 925.8251376 | 1250.990751 | 13.65931261 | 0.003624792 |
| YJR055W | HIT1 | DELETION-41204 | 895.6754498 | 1220.657539 | 13.65160326 | 0.003644023 |
| YIR022W | SEC11 | DAMP-D1928 | 503.8723538 | 1071.340167 | 13.60676493 | 0.003757721 |
| YNR032W | PPG1 | DELETION | 788.9342161 | 1111.756124 | 13.56085999 | 0.003877456 |
| YHR010W | RPL27A | DELETION | 497.557647 | 817.4924008 | 13.4395786 | 0.00421065 |
| YLL038C | ENT4 | DELETION | 739.2586091 | 1059.003492 | 13.43160263 | 0.004233447 |
| YPL139C | UME1 | DELETION | 750.0370743 | 1069.603463 | 13.4241046 | 0.00425498 |
| YPR040W | TIP41 | DELETION | 1178.855949 | 1495.608722 | 13.30591235 | 0.004607788 |
| YAL041W | CDC24 | DAMP-D0104 | 1032.456239 | 1638.005029 | 13.30401881 | 0.00461365 |
| YLR305C | STT4 | DAMP-D2213 | 663.5196815 | 1249.500615 | 13.26395356 | 0.004739273 |
| YCR081W | SRB8 | DELETION | 499.9883947 | 815.511444 | 13.25425503 | 0.004770145 |
| YDL082W | RPL13A | DELETION-43133 | 1105.964474 | 1592.352451 | 13.14071912 | 0.005145317 |
| YNL199C | GCR2 | DELETION | 683.1893219 | 995.9277524 | 13.13728086 | 0.005157083 |
| YGL186C | TPN1 | DELETION | 959.1862797 | 1269.846732 | 13.04999071 | 0.005464073 |
| YDL048C | STP4 | DELETION-32422 | 860.5785631 | 1170.913529 | 13.0363179 | 0.005513626 |
| YER123W | YCK3 | DELETION-42102 | 894.3956436 | 1203.577901 | 12.98789581 | 0.005692394 |
| YNL296W | YNL296W | DELETION | 601.427542 | 908.5969296 | 12.90334069 | 0.006017134 |
| YOR135C | IRC14 | DELETION | 539.5871036 | 844.9390327 | 12.82699426 | 0.006324587 |
| YNL069C | RPL16B | DELETION-41405 | 856.0271136 | 1161.363341 | 12.82633467 | 0.006327303 |
| YER164W | CHD1 | DELETION-43222 | 1456.395676 | 2107.728902 | 12.79022021 | 0.006477664 |
| YBR141C | YBR141C | DELETION-40345 | 916.9187276 | 1221.320091 | 12.78706363 | 0.006490958 |
| YLR358C | YLR358C | DELETION-40937 | 738.3366254 | 1041.515915 | 12.73572766 | 0.006710631 |
| YDL137W | ARF2 | DELETION | 815.3188984 | 1118.12853 | 12.72019935 | 0.006778382 |
| YMR239C | RNT1 | DAMP-D1733 | 687.7603973 | 989.8776298 | 12.69111356 | 0.006906942 |
| YDR240C | SNU56 | DAMP-D0733 | 720.7690757 | 1022.745267 | 12.68518879 | 0.006933397 |
| YOR375C | GDH1 | DELETION | 710.2247397 | 1010.990874 | 12.63435766 | 0.007164129 |
| YKR020W | VPS51 | DELETION | 733.7605555 | 1176.14607 | 12.53362774 | 0.007641826 |
| YLR384C | IKI3 | DELETION | 588.5422742 | 886.7878823 | 12.52847727 | 0.007667001 |
| YMR290C | HAS1 | DAMP-D0835 | 625.9702206 | 924.1792493 | 12.52694067 | 0.007674526 |
| YLR358C | YLR358C | DELETION-31038 | 724.1046064 | 1022.181735 | 12.52139992 | 0.007701716 |
| YLR062C | BUD28 | DELETION | 486.795698 | 784.0099886 | 12.48515446 | 0.007881722 |
| YGL054C | ERV14 | DELETION | 772.8846363 | 1381.034549 | 12.43191852 | 0.008152942 |
| YHR070W | TRM5 | DAMP-D1131 | 693.0542828 | 987.5612811 | 12.37142856 | 0.008471233 |
| YJR104C | SOD1 | DELETION | 968.3933122 | 1262.338939 | 12.3478469 | 0.008598297 |
| YDL061C | RPS29B | DELETION-32814 | 741.971698 | 1034.290058 | 12.2794899 | 0.008976305 |
| YIR022W | SEC11 | DAMP-D1927 | 488.529173 | 1080.627948 | 12.27854674 | 0.008981622 |
| YER125W | RSP5 | DAMP-D2325 | 876.159019 | 1167.008665 | 12.21779326 | 0.00933015 |
| YMR239C | RNT1 | DAMP-D1734 | 683.3089187 | 974.0215656 | 12.2120383 | 0.009363782 |
| YBR191W | RPL21A | DELETION-40537 | 550.5519328 | 841.1071855 | 12.20542661 | 0.009402555 |
| YDR168W | CDC37 | DAMP-D0322 | 923.6747446 | 1212.076205 | 12.1149517 | 0.009947698 |
| YDR450W | RPS18A | DELETION-30820 | 653.2311719 | 941.0736905 | 12.0914721 | 0.010093695 |
| YLR441C | RPS1A | DELETION | 829.3966439 | 1117.121934 | 12.08654766 | 0.010124557 |
| YOR265W | RBL2 | DELETION-33018 | 927.6282013 | 1214.767059 | 12.06191326 | 0.010280205 |
| YPL226W | NEW1 | DELETION | 691.5659362 | 977.6163908 | 12.01619243 | 0.010574738 |
| YDL029W | ARP2 | DAMP-D2119 | 819.0578877 | 1104.562613 | 11.99326785 | 0.010725224 |
| YOR304W | ISW2 | DELETION | 806.0040541 | 1091.454068 | 11.99096957 | 0.010740415 |
| YLR175W | CBF5 | DAMP-D1338 | 660.888017 | 1146.330505 | 11.98985966 | 0.010747759 |
| YNL231C | PDR16 | DELETION | 759.4243334 | 1043.629073 | 11.93865904 | 0.011091394 |
| YPL128C | TBF1 | DAMP-D1406 | 763.0967681 | 1232.18324 | 11.93095767 | 0.011143918 |
| YNL037C | IDH1 | DELETION | 598.9623603 | 882.8925533 | 11.92712608 | 0.011170132 |
| YDL056W | MBP1 | DELETION-32802 | 700.53274 | 984.140906 | 11.91359861 | 0.011263119 |
| YGL171W | ROK1 | DAMP-D0311 | 1095.210078 | 1528.217521 | 11.90644401 | 0.011312576 |
| YPL137C | GIP3 | DELETION-30604 | 888.4726733 | 1171.505431 | 11.88942727 | 0.011430984 |
| YBL023C | MCM2 | DAMP | 1011.964095 | 1553.324177 | 11.88131428 | 0.011487823 |
| YOR132W | VPS17 | DELETION | 637.765606 | 1095.461386 | 11.85306369 | 0.011687706 |
| YDL133W | YDL133W | DELETION | 789.7871878 | 1071.233119 | 11.82276909 | 0.011905474 |
| YDR240C | SNU56 | DAMP-D0734 | 721.916071 | 1001.525448 | 11.74562048 | 0.012476373 |
| YAR018C | KIN3 | DELETION | 766.3577026 | 1045.405106 | 11.72201353 | 0.012655843 |
| YBR134W | YBR134W | DELETION-30430 | 965.8411429 | 1244.690217 | 11.71368224 | 0.012719725 |
| YGR099W | TEL2 | DAMP-D0723 | 880.8290333 | 1158.65823 | 11.67084 | 0.013052752 |
| YDL120W | YFH1 | DAMP-D2147 | 640.9059807 | 918.298014 | 11.65247598 | 0.013197848 |
| YJL129C | TRK1 | DELETION-22510 | 544.4339132 | 821.4920776 | 11.63845107 | 0.013309618 |
| YMR029C | FAR8 | DELETION | 704.8352174 | 981.685203 | 11.62970605 | 0.013379734 |
| YOR117W | RPT5 | DAMP-D3047 | 650.4307132 | 1368.75308 | 11.62102354 | 0.013449671 |
| YDR168W | CDC37 | DAMP-D0321 | 996.0961703 | 1272.677291 | 11.61841178 | 0.013470771 |
| YBL033C | RIB1 | DELETION-41004 | 969.3074114 | 1245.738739 | 11.61211937 | 0.013521729 |
| YLR163C | MAS1 | DAMP-D1334 | 808.0122423 | 1084.286792 | 11.60553357 | 0.013575244 |
| YGL078C | DBP3 | DELETION | 667.1998753 | 941.9387028 | 11.5410221 | 0.014109431 |
| YDL032W | YDL032W | DELETION-41917 | 766.0016345 | 1040.132256 | 11.51547306 | 0.014326066 |
| YER111C | SWI4 | DELETION-41706 | 976.3508602 | 1249.499264 | 11.47421278 | 0.014682116 |
| YBR085C-A | YBR085C-A | DELETION | 789.390973 | 1061.018175 | 11.41031126 | 0.015248946 |
| YOL010W | RCL1 | DAMP | 752.1513242 | 1245.815434 | 11.38178208 | 0.015508172 |
| YIL063C | YRB2 | DAMP | 1140.947028 | 1767.443148 | 11.36300457 | 0.0156809 |
| YGR151C | YGR151C | DELETION | 807.9712554 | 1078.444773 | 11.36184819 | 0.015691592 |
| YFL024C | EPL1 | DAMP-D2717 | 624.5962451 | 1392.503105 | 11.32274561 | 0.016056942 |
| YBR265W | TSC10 | DAMP-D1703 | 835.4188141 | 1440.10346 | 11.31283087 | 0.016150759 |
| YER166W | DNF1 | DELETION-42926 | 992.9425871 | 1683.64675 | 11.27248401 | 0.016537525 |
| YHL001W | RPL14B | DELETION | 938.188827 | 1206.294946 | 11.26240028 | 0.016635449 |
| YNL293W | MSB3 | DELETION | 791.7037424 | 1183.932015 | 11.25980415 | 0.016660743 |
| YNL067W-B | YNL067W-B | DELETION | 891.1425144 | 1157.954178 | 11.20802374 | 0.017172331 |
| YDR061W | YDR061W | DELETION | 939.3735113 | 1205.783012 | 11.19113002 | 0.017342196 |
| YNL111C | CYB5 | DELETION-31828 | 799.1759223 | 1064.979281 | 11.16566763 | 0.017601003 |
| YER125W | RSP5 | DAMP-D2326 | 887.3599633 | 1153.153098 | 11.16523815 | 0.017605397 |
| YBR283C | SSH1 | DELETION | 732.2907297 | 996.8018406 | 11.11138387 | 0.018164084 |
| YBL014C | RRN6 | DAMP-D0128 | 881.6230802 | 1368.03968 | 11.07647776 | 0.018534441 |
| YML065W | ORC1 | DAMP-D3005 | 831.3489751 | 1484.373798 | 11.06159729 | 0.018694322 |
| YHR070W | TRM5 | DAMP-D1132 | 703.9223473 | 966.915999 | 11.04763959 | 0.018845383 |
| YDR443C | SSN2 | DELETION-30804 | 831.114065 | 1093.905457 | 11.03914323 | 0.01893786 |
| YPL137C | GIP3 | DELETION-40503 | 1029.580905 | 1291.807109 | 11.01540124 | 0.019198381 |
| YDL119C | YDL119C | DELETION | 765.464621 | 1182.294285 | 10.99213038 | 0.019456766 |
| YKL054C | DEF1 | DELETION | 580.6244392 | 841.7321709 | 10.96841728 | 0.019723182 |
| YGR271C-A | EFG1 | DELETION | 538.0134567 | 798.9781091 | 10.96240691 | 0.019791212 |
| YDR358W | GGA1 | DELETION | 798.5377284 | 1059.471157 | 10.96109527 | 0.019806086 |
| YGL066W | SGF73 | DELETION | 765.5045179 | 1026.098029 | 10.9468163 | 0.019968635 |
| YNL143C | YNL143C | DELETION | 722.6071979 | 982.9623482 | 10.93680341 | 0.020083314 |
| YIL026C | IRR1 | DAMP-D1520 | 814.2126517 | 1073.473521 | 10.89083567 | 0.020617195 |
| YGL001C | ERG26 | DAMP-D2746 | 816.9571288 | 1257.612664 | 10.87418365 | 0.020813627 |
| YJL154C | VPS35 | DELETION | 653.3654409 | 912.1241584 | 10.86974166 | 0.0208663 |
| YGL194C | HOS2 | DELETION | 653.932526 | 912.2551848 | 10.85142403 | 0.021084743 |
| YML023C | NSE5 | DAMP-D2829 | 728.5773651 | 1394.569226 | 10.82082564 | 0.021454081 |
| YMR030W | RSF1 | DELETION | 773.2311508 | 1030.210708 | 10.79500405 | 0.021770132 |
| YLR039C | RIC1 | DELETION | 767.0493049 | 1023.51921 | 10.77359494 | 0.022035239 |
| YMR100W | MUB1 | DELETION | 804.8560862 | 1061.160445 | 10.7666408 | 0.022121954 |
| YCL054W | SPB1 | DAMP-D1720 | 623.2929007 | 1055.434567 | 10.74401685 | 0.022406121 |
| YER021W | RPN3 | DAMP | 630.9779676 | 1070.514375 | 10.73599894 | 0.022507588 |
| YER123W | YCK3 | DELETION-42402 | 864.1356531 | 1330.160382 | 10.73586199 | 0.022509324 |
| YDL195W | SEC31 | DAMP-D2528 | 824.1184142 | 1199.634574 | 10.70128798 | 0.022951478 |
| YMR080C | NAM7 | DELETION | 742.9749829 | 997.2115985 | 10.6797806 | 0.023230318 |
| YBR043C | QDR3 | DELETION | 988.6900909 | 1242.07461 | 10.64398637 | 0.023700908 |
| YOR376W | YOR376W | DELETION | 703.5509199 | 956.5203068 | 10.62654781 | 0.023933155 |
| YDL048C | STP4 | DELETION-42321 | 910.9348403 | 1163.401862 | 10.60544482 | 0.024216842 |
| YJL011C | RPC17 | DAMP-D2303 | 780.1050394 | 1172.277194 | 10.60490481 | 0.02422414 |
| YBR018C | GAL7 | DELETION | 844.330425 | 1095.463157 | 10.549395 | 0.024984515 |
| YFR027W | ECO1 | DAMP | 526.7364826 | 982.4163062 | 10.51471093 | 0.025470037 |
| YBR099C | YBR099C | DELETION-42531 | 826.5587023 | 1076.83706 | 10.51350509 | 0.025487062 |
| YER044C | ERG28 | DELETION-42024 | 914.0542598 | 1164.308755 | 10.51250269 | 0.025501223 |
| YHR129C | ARP1 | DELETION | 788.2778171 | 1038.132404 | 10.49570363 | 0.025739556 |
| YAL012W | CYS3 | DELETION | 706.009355 | 955.8599936 | 10.49553778 | 0.025741918 |
| YLR261C | VPS63 | DELETION | 665.6852495 | 915.4529733 | 10.49205476 | 0.025791577 |
| YOL015W | IRC10 | DELETION | 768.3755972 | 1018.12878 | 10.49144394 | 0.025800294 |
| YPL249C-A | RPL36B | DELETION | 842.5404806 | 1495.710705 | 10.47667025 | 0.026011912 |
| YGL223C | COG1 | DELETION-40344 | 363.331116 | 612.3839088 | 10.46202247 | 0.026223207 |
| YNR011C | PRP2 | DAMP-D2227 | 684.9409939 | 1207.082474 | 10.4618451 | 0.026225775 |
| YDR082W | STN1 | DAMP-D2935 | 873.1140498 | 1121.972917 | 10.45387618 | 0.026341358 |
| YPR042C | PUF2 | DELETION | 1067.147273 | 1315.619328 | 10.43762729 | 0.026578401 |
| YOL004W | SIN3 | DELETION | 493.5859516 | 1166.077008 | 10.41782398 | 0.026869786 |
| YPL128C | TBF1 | DAMP-D0616 | 610.6162186 | 1190.014127 | 10.41728083 | 0.026877816 |
| YDL133C-A | RPL41B | DELETION | 1034.212525 | 1281.361039 | 10.38202898 | 0.027403474 |
| YNL059C | ARP5 | DELETION | 1073.930166 | 1320.603637 | 10.36207369 | 0.02770495 |
| YDL208W | NHP2 | DAMP-D2539 | 677.1210381 | 923.5313046 | 10.35101721 | 0.027873215 |
| YMR197C | VTI1 | DAMP | 639.6553061 | 1289.576988 | 10.33860857 | 0.028063107 |
| YNL057W | YNL057W | DELETION | 1064.823542 | 1769.617566 | 10.33101299 | 0.028179893 |
| YNR046W | TRM112 | DAMP-D2239 | 648.7412078 | 894.4987671 | 10.32359878 | 0.028294294 |
| YNR011C | PRP2 | DAMP-D2427 | 712.2408566 | 1397.686683 | 10.29927654 | 0.028672399 |
| YNL021W | HDA1 | DELETION | 871.5047741 | 1116.407731 | 10.28769927 | 0.028853898 |
| YPL107W | YPL107W | DELETION | 926.0776276 | 1170.505959 | 10.26776156 | 0.029168785 |
| YBL033C | RIB1 | DELETION-41104 | 917.1565747 | 1161.305884 | 10.25604061 | 0.029355277 |
| YGR211W | ZPR1 | DAMP-D0747 | 605.3412276 | 849.2876262 | 10.2475169 | 0.029491541 |
| YER164W | CHD1 | DELETION-42922 | 989.1432619 | 1655.145676 | 10.24639625 | 0.029509496 |
| YIR012W | SQT1 | DAMP-D0934 | 738.2792335 | 981.8077926 | 10.22996461 | 0.029773854 |
| YHR205W | SCH9 | DELETION | 723.540034 | 1120.808713 | 10.22758711 | 0.029812273 |
| YIR012W | SQT1 | DAMP-D0933 | 731.9652842 | 975.1372318 | 10.21498435 | 0.030016634 |
| YBR095C | RXT2 | DELETION-42523 | 762.4427683 | 1005.466866 | 10.2087736 | 0.030117787 |
| YLR089C | ALT1 | DELETION | 530.4178402 | 773.3121171 | 10.20332017 | 0.030206848 |
| YGL058W | RAD6 | DELETION | 541.2594498 | 783.5871583 | 10.17952019 | 0.030598181 |
| YLR402W | YLR402W | DELETION | 619.7827999 | 861.2238197 | 10.14227283 | 0.03121936 |
| YER133W | GLC7 | DAMP-D2333 | 658.189354 | 899.4806423 | 10.13598303 | 0.031325317 |
| YDL017W | CDC7 | DAMP-D2111 | 759.2879982 | 1000.542791 | 10.13444994 | 0.03135119 |
| YMR220W | ERG8 | DAMP-D0803 | 1001.883246 | 1476.801938 | 10.1252311 | 0.031507157 |
| YLR129W | DIP2 | DAMP | 636.2513316 | 876.9049149 | 10.10919479 | 0.031780049 |
| YHR013C | ARD1 | DELETION | 722.0272047 | 962.5920719 | 10.10546807 | 0.031843757 |
| YOR327C | SNC2 | DELETION | 859.5098827 | 1099.020863 | 10.06119718 | 0.032608974 |
| YCR053W | THR4 | DELETION-30242 | 680.9457374 | 920.3751085 | 10.05776901 | 0.032668882 |
| YMR052C-A | YMR052C-A | DELETION-40808 | 824.8935257 | 1063.984423 | 10.04355066 | 0.032918354 |
| YMR230W | RPS10B | DELETION | 694.5779867 | 933.0645288 | 10.01816335 | 0.033367851 |
| YML101C-A | YML101C-A | DELETION | 798.0006475 | 1035.96005 | 9.996019689 | 0.033764195 |
| YLR217W | YLR217W | DELETION | 782.2265945 | 1019.958102 | 9.986446423 | 0.033936787 |
| YJR055W | HIT1 | DELETION-32614 | 573.6819828 | 811.1545587 | 9.975569429 | 0.034133798 |
| YDR002W | YRB1 | DAMP-D2908 | 657.3706081 | 1087.436845 | 9.965774654 | 0.034312043 |
| YBR148W | YSW1 | DELETION-30818 | 832.0626308 | 1068.374522 | 9.926812263 | 0.035028976 |
| YNL145W | MFA2 | DELETION | 796.7568152 | 1033.003027 | 9.924053231 | 0.035080225 |
| YKL007W | CAP1 | DELETION | 779.4337828 | 1015.667383 | 9.923523464 | 0.035090073 |
| YOL151W | GRE2 | DELETION-33002 | 778.6749967 | 1014.795541 | 9.918774304 | 0.03517846 |
| YCL054W | SPB1 | DAMP-D1719 | 621.5140767 | 1054.967343 | 9.918191681 | 0.035189316 |
| YOL072W | THP1 | DELETION | 542.3735282 | 778.30625 | 9.910884389 | 0.03532572 |
| YMR143W | RPS16A | DELETION-42427 | 560.8233906 | 795.8398892 | 9.872396376 | 0.036051639 |
| YHR178W | STB5 | DELETION | 887.3385934 | 1122.334537 | 9.871532903 | 0.03606807 |
| YMR017W | SPO20 | DELETION | 774.0040203 | 1008.176978 | 9.836961537 | 0.036731179 |
| YDR082W | STN1 | DAMP-D2936 | 889.3020786 | 1123.143951 | 9.823053565 | 0.03700086 |
| YNR046W | TRM112 | DAMP-D2439 | 631.7272152 | 865.429112 | 9.817173572 | 0.037115382 |
| YER098W | UBP9 | DELETION | 885.9873001 | 1119.376685 | 9.804045807 | 0.037372154 |
| YOR128C | ADE2 | DELETION | 818.0365592 | 1051.095968 | 9.790184413 | 0.037644915 |
| YLR005W | SSL1 | DAMP-D1029 | 621.3135862 | 1312.586981 | 9.780543436 | 0.037835626 |
| YPR139C | VPS66 | DELETION | 488.393912 | 863.8797695 | 9.743231719 | 0.038581459 |
| YKL037W | AIM26 | DELETION | 770.6178257 | 1002.44643 | 9.738481731 | 0.038677299 |
| YLR287-A | 0 | DELETION | 732.7058419 | 964.0419619 | 9.717793802 | 0.039097077 |
| YJR055W | HIT1 | DELETION-30712 | 580.3118957 | 811.0935769 | 9.694503349 | 0.039574287 |
| YJR033C | RAV1 | DELETION | 1042.159034 | 1272.706962 | 9.684684037 | 0.039776956 |
| YOR083W | WHI5 | DELETION | 826.7207969 | 1057.212442 | 9.682319749 | 0.039825886 |
| YHR036W | BRL1 | DAMP | 701.9377044 | 931.8209092 | 9.656760835 | 0.040358107 |
| YER150W | SPI1 | DELETION-42822 | 918.4833935 | 1148.334487 | 9.655411929 | 0.040386363 |
| YAL047C | SPC72 | DELETION | 477.5717748 | 706.2312895 | 9.60535698 | 0.041446784 |
| YOR279C | RFM1 | DELETION | 702.7736555 | 931.159903 | 9.593877775 | 0.041693269 |
| YNL141W | AAH1 | DELETION | 952.296466 | 1180.447788 | 9.584009214 | 0.04190616 |
| YLR175W | CBF5 | DAMP-D1337 | 671.9122255 | 1152.679624 | 9.581296249 | 0.041964847 |
| YDL120W | YFH1 | DAMP-D2148 | 817.5314477 | 1045.192495 | 9.563414099 | 0.042353417 |
| YBR144C | YBR144C | DELETION-30802 | 730.602581 | 958.1953073 | 9.560544134 | 0.042416063 |
| YDL017W | CDC7 | DAMP-D2112 | 766.3592757 | 993.7703949 | 9.552915316 | 0.042582965 |
| YDR226W | ADK1 | DELETION | 366.2032132 | 593.2982743 | 9.539638586 | 0.042874757 |
| YDL032W | YDL032W | DELETION-32018 | 568.5132239 | 795.3630643 | 9.529337541 | 0.043102313 |
| YDR195W | REF2 | DELETION | 687.1849237 | 913.7239864 | 9.516282624 | 0.043392169 |
| YAL041W | CDC24 | DAMP-D0103 | 1141.334057 | 1723.686426 | 9.512179693 | 0.043483605 |
| YGL244W | RTF1 | DELETION | 646.5788264 | 872.7865255 | 9.502362948 | 0.043703037 |
| YLR458W | YLR458W | DAMP | 829.3105374 | 1055.503726 | 9.501753403 | 0.043716693 |
| YGR211W | ZPR1 | DAMP-D0748 | 847.5478978 | 1073.378631 | 9.486527648 | 0.044058972 |
| YML012C-A | YML012C-A | DELETION | 615.223539 | 840.7709808 | 9.474627358 | 0.044328067 |
| YML076C | WAR1 | DELETION-31802 | 752.8924533 | 978.2288541 | 9.465762106 | 0.044529432 |
| YPL152W | RRD2 | DELETION | 763.3766153 | 988.6602518 | 9.463545625 | 0.044579897 |
| YNL235C | YNL235C | DELETION | 757.2940248 | 982.5335115 | 9.461691012 | 0.044622161 |
| YIL037C | PRM2 | DELETION | 729.3979443 | 954.2084586 | 9.443671064 | 0.045034567 |
| YML023C | NSE5 | DAMP-D2629 | 704.6230168 | 1349.240881 | 9.431517575 | 0.045314524 |
| YDR360W | OPI7 | DELETION | 695.4243261 | 919.9150191 | 9.430236248 | 0.045344125 |
| YGR149W | YGR149W | DELETION | 941.0482006 | 1164.97087 | 9.406375129 | 0.045898342 |
| YGL225W | VRG4 | DAMP-D0320 | 969.2617346 | 1392.872275 | 9.396200461 | 0.046136396 |
| YGR090W | UTP22 | DAMP-D0711 | 808.158357 | 1031.215931 | 9.370034843 | 0.046753366 |
| YFL037W | TUB2 | DAMP | 809.4172493 | 1032.188491 | 9.358006838 | 0.0470393 |
| YPR159W | KRE6 | DELETION | 711.2241803 | 1095.404124 | 9.356366385 | 0.047078411 |
| YNL127W | FAR11 | DELETION | 818.3845436 | 1041.101385 | 9.355721638 | 0.047093791 |
| YDL208W | NHP2 | DAMP-D2540 | 695.6716933 | 918.1491723 | 9.345666673 | 0.047334183 |
| YLR106C | MDN1 | DAMP | 717.6586103 | 939.6565121 | 9.325520952 | 0.047818927 |
| YDR136C | VPS61 | DELETION | 643.540798 | 865.2175585 | 9.312030689 | 0.048145856 |
| YLL040C | VPS13 | DELETION | 678.6983786 | 1158.126181 | 9.308754062 | 0.048225546 |
| YMR244W | YMR244W | DELETION | 742.3073101 | 963.6275648 | 9.29705486 | 0.048510984 |
| YOR082C | YOR082C | DELETION | 791.6344653 | 1012.94415 | 9.296610854 | 0.048521845 |
| YCL050C | APA1 | DELETION | 803.0903139 | 1023.596034 | 9.262838519 | 0.049353951 |
| YOR329C | SCD5 | DAMP-D0646 | 760.8940063 | 981.0730738 | 9.249116729 | 0.049695442 |
|  |  |  |  |  |  |  |
| **Enhancers** | | | | | | |
| **ORF** | **Gene name** | **Mutation** | **WT.mean** | **MYR.mean** | **T-score** | **Significance** |
| YNR075W | COS10 | DELETION | 1163.459773 | 955.3174802 | -8.743484949 | 0.04927388 |
| YBL063W | KIP1 | DELETION | 1056.524506 | 848.2612698 | -8.74856543 | 0.049143669 |
| YER162C | RAD4 | DELETION-42914 | 1236.956951 | 742.9698906 | -8.764193502 | 0.048744935 |
| YDR501W | PLM2 | DELETION-32024 | 889.1460098 | 680.139173 | -8.779802062 | 0.048349413 |
| YNR030W | ALG12 | DELETION | 975.4069925 | 766.3315589 | -8.782683624 | 0.04827669 |
| YIL107C | PFK26 | DELETION | 870.4077868 | 661.2519595 | -8.786060744 | 0.048191577 |
| YMR208W | ERG12 | DAMP-D0239 | 734.6829134 | 524.8250459 | -8.815551519 | 0.047453669 |
| YDR291W | HRQ1 | DELETION | 960.2295605 | 749.8209872 | -8.838685153 | 0.046881497 |
| YLR177W | YLR177W | DELETION | 884.0485205 | 673.6243061 | -8.83934219 | 0.046865332 |
| YPL149W | ATG5 | DELETION | 1037.418179 | 826.9351418 | -8.841813162 | 0.046804579 |
| YBR222C | PCS60 | DELETION | 976.8384619 | 765.9496413 | -8.858859018 | 0.046387279 |
| YGL123W | RPS2 | DAMP-D3146 | 894.7661834 | 683.7811772 | -8.862899513 | 0.046288825 |
| YGR092W | DBF2 | DELETION | 668.5452108 | 457.5461863 | -8.86348838 | 0.04627449 |
| YIL029C | YIL029C | DELETION | 913.7309331 | 702.6677847 | -8.866182051 | 0.046208969 |
| YHR087W | RTC3 | DELETION | 937.1634822 | 726.0790942 | -8.867074267 | 0.046187283 |
| YJR127C | RSF2 | DELETION | 840.1407777 | 628.78845 | -8.878329678 | 0.045914455 |
| YNL255C | GIS2 | DELETION | 996.1032593 | 784.3611318 | -8.894704095 | 0.045519969 |
| YAL051W | OAF1 | DELETION | 1049.599318 | 837.7420392 | -8.899541268 | 0.045403981 |
| YGR191W | HIP1 | DAMP-D0740 | 796.0649614 | 583.3341692 | -8.936235176 | 0.044532208 |
| YHL022C | SPO11 | DELETION | 824.3395924 | 611.3686957 | -8.946321307 | 0.044295071 |
| YLR026C | SED5 | DAMP-D1401 | 1215.292357 | 864.9733728 | -8.952283344 | 0.044155398 |
| YBR234C | ARC40 | DAMP-D1328 | 875.6509764 | 662.4224758 | -8.957142539 | 0.044041837 |
| YHR008C | SOD2 | DELETION | 785.8415049 | 572.61089 | -8.957231357 | 0.044039763 |
| YBR271W | YBR271W | DELETION | 916.1942232 | 702.8186539 | -8.963320487 | 0.043897812 |
| YMR152W | YIM1 | DELETION | 1062.392595 | 848.8827515 | -8.968960976 | 0.043766664 |
| YBL106C | SRO77 | DELETION | 885.8263972 | 671.9808999 | -8.983060872 | 0.043440272 |
| YMR208W | ERG12 | DAMP-D0439 | 938.1887515 | 724.0688656 | -8.994587184 | 0.043174984 |
| YKL173W | SNU114 | DAMP-D1306 | 857.9742 | 643.3311424 | -9.016564187 | 0.042672958 |
| YDR009W | GAL3 | DELETION | 931.5182908 | 716.8628255 | -9.0170854 | 0.042661112 |
| YDR053W | YDR053W | DAMP | 785.0092343 | 570.2265104 | -9.022431181 | 0.042539774 |
| YNL052W | COX5A | DELETION | 556.6639308 | 341.8745035 | -9.022712775 | 0.042533391 |
| YDL159W | STE7 | DELETION | 979.727813 | 764.884396 | -9.024980734 | 0.042482008 |
| YKL222C | YKL222C | DELETION | 955.6028335 | 740.7164612 | -9.026785166 | 0.042441164 |
| YNL328C | MDJ2 | DELETION | 839.1131787 | 623.9214928 | -9.039610553 | 0.042151815 |
| YDR414C | ERD1 | DELETION | 916.407731 | 700.9574228 | -9.050474558 | 0.041908023 |
| YJL095W | BCK1 | DELETION | 762.6010648 | 547.0585392 | -9.054348359 | 0.041821383 |
| YDR283C | GCN2 | DELETION-41148 | 1127.314178 | 911.2656146 | -9.075605621 | 0.041348644 |
| YLR066W | SPC3 | DAMP-D1409 | 912.9329366 | 696.7907902 | -9.079536778 | 0.041261716 |
| YLR066W | SPC3 | DAMP-D1609 | 898.373949 | 682.196149 | -9.081034492 | 0.041228639 |
| YPR076W | YPR076W | DELETION | 864.0951301 | 647.8876361 | -9.082281853 | 0.041201108 |
| YDR508C | GNP1 | DELETION-32048 | 983.1568042 | 766.7148497 | -9.092130892 | 0.040984273 |
| YMR068W | AVO2 | DELETION | 813.4851328 | 596.6938425 | -9.106805531 | 0.040662991 |
| YER177W | BMH1 | DELETION-40112 | 1250.299022 | 394.7518495 | -9.135914465 | 0.040032005 |
| YPL063W | TIM50 | DAMP-D0236 | 851.5691812 | 634.008831 | -9.139111617 | 0.03996321 |
| YML098W | TAF13 | DAMP-D1710 | 791.9922173 | 574.0785502 | -9.15395349 | 0.039645161 |
| YBR242W | YBR242W | DELETION | 882.9153838 | 664.1948679 | -9.187846989 | 0.038926899 |
| YDL115C | IWR1 | DELETION-30624 | 725.5636574 | 506.7079412 | -9.19352638 | 0.038807631 |
| YLR127C | APC2 | DAMP-D1641 | 872.0663579 | 653.0016859 | -9.202304041 | 0.038623909 |
| YPL134C | ODC1 | DELETION | 977.924766 | 758.8152592 | -9.204187425 | 0.038584585 |
| YBR153W | RIB7 | DAMP-D0948 | 912.3716916 | 342.2946591 | -9.206334551 | 0.038539796 |
| YHR061C | GIC1 | DELETION | 963.1379747 | 743.7729481 | -9.214921108 | 0.038361122 |
| YMR166C | YMR166C | DELETION | 907.6186226 | 687.7457303 | -9.23625515 | 0.037920235 |
| YAR029W | YAR029W | DELETION | 1040.380617 | 820.5051336 | -9.236364007 | 0.037917996 |
| YHR039C-A | VMA10 | DELETION | 328.823706 | 108.6577794 | -9.248564716 | 0.037667813 |
| YGR039W | YGR039W | DELETION | 1085.999305 | 865.7092164 | -9.25378043 | 0.037561291 |
| YIR004W | DJP1 | DELETION-40936 | 932.333099 | 712.0203549 | -9.254732114 | 0.037541882 |
| YOR177C | MPC54 | DELETION | 1020.594825 | 800.0180472 | -9.265823454 | 0.037316311 |
| YGR169C | PUS6 | DELETION | 1100.360148 | 879.1186218 | -9.293747679 | 0.036753504 |
| YBR238C | YBR238C | DELETION | 992.718518 | 771.1869625 | -9.305931027 | 0.036510226 |
| YGL238W | CSE1 | DAMP-D0328 | 1040.195387 | 818.429037 | -9.315794084 | 0.036314285 |
| YPL182C | YPL182C | DELETION | 885.2736918 | 663.4309093 | -9.319004816 | 0.036250694 |
| YBR102C | EXO84 | DAMP-D0923 | 864.7489286 | 642.3663578 | -9.341679837 | 0.035804295 |
| YGR217W | CCH1 | DELETION | 803.8215747 | 581.2280195 | -9.350542711 | 0.035631092 |
| YOR326W | MYO2 | DAMP-D0204 | 947.7846822 | 480.9933178 | -9.365667528 | 0.035337165 |
| YCR012W | PGK1 | DAMP | 986.5447479 | 763.3681219 | -9.375035909 | 0.035156145 |
| YKL137W | CMC1 | DELETION-22107 | 642.3681761 | 419.1395 | -9.377222392 | 0.035114011 |
| YMR202W | ERG2 | DELETION-12303 | 1123.482551 | 899.7618533 | -9.397890864 | 0.03471785 |
| YNL307C | MCK1 | DELETION | 867.3899957 | 643.555064 | -9.402689524 | 0.03462642 |
| YER117W | RPL23B | DELETION-42026 | 851.0150092 | 626.6055383 | -9.426824333 | 0.034169686 |
| YOR026W | BUB3 | DELETION-11328 | 610.4979462 | 385.8604389 | -9.436403515 | 0.03398984 |
| YBR231C | SWC5 | DELETION | 779.3400283 | 554.6286937 | -9.439504796 | 0.033931788 |
| YHR026W | PPA1 | DELETION | 224.7174867 | 0 | -9.439763229 | 0.033926954 |
| YJL152W | YJL152W | DELETION | 937.1500089 | 711.9653974 | -9.459385855 | 0.03356165 |
| YDL185W | TFP1 | DELETION-42616 | 507.695533 | 282.44175 | -9.462291563 | 0.033507843 |
| YKL136W | YKL136W | DELETION | 994.6387831 | 769.1020659 | -9.474176848 | 0.033288522 |
| YOL097W-A | YOL097W-A | DELETION | 986.9716557 | 760.9749637 | -9.493499122 | 0.032934588 |
| YEL008W | YEL008W | DELETION | 934.2399988 | 708.158685 | -9.497053852 | 0.032869827 |
| YGL065C | ALG2 | DAMP | 622.0408392 | 395.3817505 | -9.521324587 | 0.03243056 |
| YDL026W | YDL026W | DELETION-41909 | 1222.53731 | 797.5488176 | -9.539299699 | 0.032108486 |
| YDR430C | CYM1 | DELETION | 998.9150388 | 771.6983067 | -9.54474965 | 0.032011378 |
| YBR066C | NRG2 | DELETION | 846.8933969 | 619.4187511 | -9.555583895 | 0.031819079 |
| YDR410C | STE14 | DELETION | 812.0765818 | 584.0961226 | -9.576831723 | 0.031444823 |
| YBR109C | CMD1 | DAMP-D0927 | 942.5583028 | 714.5243355 | -9.579079452 | 0.031405454 |
| YDR283C | GCN2 | DELETION-41048 | 1145.14048 | 917.0912843 | -9.57971914 | 0.031394257 |
| YPL018W | CTF19 | DELETION | 1063.139155 | 834.5030681 | -9.604372848 | 0.030965343 |
| YPR120C | CLB5 | DELETION | 980.1513024 | 750.8742013 | -9.631300082 | 0.030502631 |
| YGL238W | CSE1 | DAMP-D0327 | 1033.705541 | 804.3745375 | -9.633564364 | 0.030463994 |
| YLR418C | CDC73 | DELETION | 583.4871499 | 354.0562452 | -9.637760949 | 0.030392496 |
| YLR446W | YLR446W | DELETION | 904.4529696 | 674.9798183 | -9.639535614 | 0.030362304 |
| YCR028C | FEN2 | DELETION | 464.1242047 | 234.406237 | -9.649819675 | 0.030187852 |
| YIL152W | YIL152W | DELETION | 970.8328548 | 740.871539 | -9.66004206 | 0.0300153 |
| YML048W | GSF2 | DELETION | 852.5580324 | 622.182134 | -9.677457535 | 0.029723282 |
| YJR090C | GRR1 | DELETION-41924 | 588.2025702 | 188.9945202 | -9.68551313 | 0.029589037 |
| YHR115C | DMA1 | DELETION | 900.6789451 | 670.0759679 | -9.686996487 | 0.029564374 |
| YMR031W-A | YMR031W-A | DELETION | 769.9507426 | 538.5697892 | -9.719677131 | 0.029025478 |
| YHR059W | FYV4 | DELETION | 824.896856 | 593.3770235 | -9.725511053 | 0.028930172 |
| YIL042C | PKP1 | DELETION | 989.304196 | 757.5858924 | -9.733848282 | 0.02879444 |
| YDL185W | TFP1 | DELETION-42716 | 557.7959279 | 325.1930719 | -9.771005893 | 0.02819615 |
| YLL050C | COF1 | DAMP-D1025 | 944.9895589 | 712.3133806 | -9.774085965 | 0.028147041 |
| YNL233W | BNI4 | DELETION | 845.8191749 | 613.1087346 | -9.775525215 | 0.028124118 |
| YPL218W | SAR1 | DAMP-D1430 | 800.8385394 | 567.8886101 | -9.785585485 | 0.027964341 |
| YJL128C | PBS2 | DELETION-40943 | 771.2354404 | 301.3299646 | -9.786613267 | 0.027948062 |
| YDL179W | PCL9 | DELETION | 879.7090224 | 646.0550604 | -9.815159963 | 0.027499163 |
| YMR185W | YMR185W | DAMP-D0431 | 984.8100711 | 751.0055447 | -9.821484764 | 0.027400551 |
| YKL008C | LAC1 | DELETION | 954.3783872 | 719.9051559 | -9.849575215 | 0.026966261 |
| YBL068W | PRS4 | DELETION | 951.6900938 | 717.124746 | -9.853444772 | 0.026906904 |
| YKR010C | TOF2 | DELETION | 866.9025556 | 631.984717 | -9.868251943 | 0.026680811 |
| YML004C | GLO1 | DELETION | 894.2357095 | 658.9194582 | -9.884988166 | 0.026427241 |
| YKL182W | FAS1 | DAMP-D0445 | 888.127379 | 652.6376475 | -9.892275587 | 0.026317481 |
| YHL032C | GUT1 | DELETION | 894.7961738 | 659.2236563 | -9.895753202 | 0.026265242 |
| YLR443W | ECM7 | DELETION | 964.9534451 | 729.3053776 | -9.89892685 | 0.026217647 |
| YMR115W | MGR3 | DELETION | 725.2908365 | 488.8075343 | -9.934012761 | 0.025696416 |
| YJL187C | SWE1 | DELETION | 962.0585176 | 725.3707387 | -9.942602262 | 0.025570186 |
| YPL165C | SET6 | DELETION-11801 | 1059.680296 | 822.2294622 | -9.974656122 | 0.025103851 |
| YIL028W | YIL028W | DELETION | 919.3383791 | 680.1339507 | -10.04831978 | 0.024059974 |
| YML097C | VPS9 | DELETION | 634.4126085 | 395.1192373 | -10.05205602 | 0.024008046 |
| YHL040C | ARN1 | DELETION | 863.754504 | 624.2525488 | -10.06081807 | 0.023886649 |
| YLR078C | BOS1 | DAMP-D1617 | 831.9903278 | 591.7687032 | -10.0910494 | 0.023471875 |
| YHR025W | THR1 | DELETION | 575.0915055 | 334.1636707 | -10.12071535 | 0.02307095 |
| YBR082C | UBC4 | DELETION-32244 | 730.473444 | 489.2651771 | -10.13249552 | 0.022913402 |
| YBR218C | PYC2 | DELETION | 906.6155313 | 665.322057 | -10.13607485 | 0.022865718 |
| YPL127C | HHO1 | DELETION | 996.7870299 | 755.13485 | -10.15114309 | 0.02266592 |
| YOR007C | SGT2 | DELETION | 970.7912543 | 729.091898 | -10.15312484 | 0.022639757 |
| YKR070W | YKR070W | DELETION | 911.0393526 | 668.9074843 | -10.17129348 | 0.022401109 |
| YIL168W | YIL168W | DELETION | 955.8096712 | 713.2790897 | -10.18804232 | 0.02218305 |
| YDL204W | RTN2 | DELETION | 1087.636499 | 844.6205863 | -10.20842975 | 0.021920113 |
| YPR060C | ARO7 | DELETION | 813.9169815 | 570.5857666 | -10.22167473 | 0.021750749 |
| YCL061C | MRC1 | DELETION-12020 | 934.5850372 | 690.9719953 | -10.2335135 | 0.021600331 |
| YBR091C | TIM12 | DAMP-D0920 | 840.8221965 | 596.7541773 | -10.25262585 | 0.02135941 |
| YJL149W | DAS1 | DELETION | 882.9469865 | 638.5105767 | -10.26810092 | 0.021166056 |
| YJR115W | YJR115W | DELETION | 878.5087096 | 633.1098676 | -10.30853005 | 0.020668091 |
| YKL201C | MNN4 | DELETION-42019 | 899.7906093 | 654.2732036 | -10.31351059 | 0.020607459 |
| YIL040W | APQ12 | DELETION | 350.9563129 | 104.6854749 | -10.34516021 | 0.02022577 |
| YGL020C | GET1 | DELETION-41008 | 774.4561342 | 296.9422092 | -10.34893942 | 0.020180608 |
| YDR085C | AFR1 | DELETION | 930.5556231 | 683.9424391 | -10.35954122 | 0.020054385 |
| YDR495C | VPS3 | DELETION-32008 | 451.0105588 | 204.2595142 | -10.36533237 | 0.019985729 |
| YPL026C | SKS1 | DELETION | 1069.588072 | 822.5462221 | -10.37754829 | 0.019841576 |
| YLR006C | SSK1 | DELETION-41336 | 830.7430252 | 443.7881376 | -10.45164654 | 0.018986489 |
| YDR001C | NTH1 | DELETION | 827.0818449 | 578.2541043 | -10.45256865 | 0.018976054 |
| YDL212W | SHR3 | DAMP-D2543 | 692.5611399 | 442.9181366 | -10.48681559 | 0.018592042 |
| YML019W | OST6 | DELETION | 1042.825186 | 792.9592212 | -10.49618159 | 0.018488211 |
| YNL297C | MON2 | DELETION | 598.1806397 | 347.728515 | -10.52080455 | 0.018217662 |
| YGL148W | ARO2 | DELETION-40810 | 920.4157694 | 669.9630019 | -10.52083155 | 0.018217367 |
| YJR083C | ACF4 | DELETION | 894.4995764 | 642.851299 | -10.57105164 | 0.017676298 |
| YDR177W | UBC1 | DAMP-D0334 | 1073.419608 | 821.2658485 | -10.59228554 | 0.017451795 |
| YNR052C | POP2 | DELETION-42819 | 672.1639852 | 188.1618087 | -10.62006593 | 0.017161853 |
| YLR078C | BOS1 | DAMP-D1417 | 842.5325959 | 587.7213305 | -10.70392006 | 0.016312149 |
| YHL036W | MUP3 | DELETION | 832.8141621 | 576.7807849 | -10.75525761 | 0.015810381 |
| YPR192W | AQY1 | DELETION | 1005.228665 | 749.1792917 | -10.75592954 | 0.015803905 |
| YNL090W | RHO2 | DELETION-32829 | 878.0947343 | 621.6043368 | -10.77445578 | 0.015626257 |
| YIR036C | IRC24 | DELETION | 1079.956579 | 823.425982 | -10.77614443 | 0.015610152 |
| YMR172W | HOT1 | DELETION | 1107.714727 | 851.0289439 | -10.7826634 | 0.015548118 |
| YGL115W | SNF4 | DELETION-33220 | 850.0964794 | 592.4379971 | -10.82352381 | 0.015164207 |
| YLR431C | ATG23 | DELETION | 1326.063379 | 905.9578046 | -10.83205938 | 0.015085072 |
| YOR164C | GET4 | DELETION | 868.9655784 | 610.50847 | -10.85707189 | 0.014855266 |
| YJL185C | YJL185C | DELETION | 962.0454175 | 703.1224756 | -10.87664028 | 0.014677636 |
| YKL221W | MCH2 | DELETION | 983.6246374 | 724.6989349 | -10.87675624 | 0.014676589 |
| YNL242W | ATG2 | DELETION | 1017.597955 | 758.1575482 | -10.89837755 | 0.014482514 |
| YOL141W | PPM2 | DELETION | 884.854512 | 624.5889825 | -10.93303868 | 0.014176114 |
| YER118C | SHO1 | DELETION-41730 | 957.1106808 | 574.6855994 | -10.93754165 | 0.014136731 |
| YKL142W | MRP8 | DELETION | 909.3949366 | 648.9557591 | -10.94033316 | 0.014112365 |
| YCL008C | STP22 | DELETION | 938.0477558 | 676.2961955 | -10.99546275 | 0.013638695 |
| YCL055W | KAR4 | DELETION | 983.9764501 | 721.3116363 | -11.03382601 | 0.013317421 |
| YHR109W | CTM1 | DELETION | 907.0235802 | 643.652528 | -11.06349315 | 0.013073579 |
| YDL172C | YDL172C | DELETION | 712.2998129 | 448.5840387 | -11.07797397 | 0.012955997 |
| YDR112W | IRC2 | DELETION | 1085.466614 | 821.3135257 | -11.09634434 | 0.01280818 |
| YDL205C | HEM3 | DAMP-D2532 | 598.3540505 | 333.4283942 | -11.12879779 | 0.012550687 |
| YDR308C | SRB7 | DAMP-D1114 | 362.6809666 | 97.63165072 | -11.13399239 | 0.012509901 |
| YKL202W | YKL202W | DELETION | 901.9813948 | 636.3401971 | -11.15885571 | 0.012316301 |
| YDL212W | SHR3 | DAMP-D2544 | 676.2106001 | 410.5384229 | -11.16015708 | 0.012306241 |
| YKL133C | YKL133C | DELETION | 875.0727134 | 608.2070968 | -11.21029018 | 0.011924207 |
| YPL264C | YPL264C | DELETION | 906.2054546 | 638.7714274 | -11.23416754 | 0.011745978 |
| YDL205C | HEM3 | DAMP-D2531 | 605.9553944 | 338.1649107 | -11.2491413 | 0.011635417 |
| YGR077C | PEX8 | DELETION | 946.6604358 | 678.8574957 | -11.24966456 | 0.01163157 |
| YDR183W | PLP1 | DELETION | 1001.665233 | 732.4867389 | -11.30744778 | 0.011213651 |
| YLR243W | YLR243W | DAMP-D2045 | 841.1519776 | 571.804268 | -11.31455608 | 0.011163173 |
| YDR395W | SXM1 | DELETION | 928.0265272 | 658.3221224 | -11.32953986 | 0.011057428 |
| YMR191W | SPG5 | DELETION-32626 | 920.4342066 | 650.5921978 | -11.33532023 | 0.011016873 |
| YOR347C | PYK2 | DELETION | 920.0751516 | 650.040847 | -11.34339804 | 0.01096042 |
| YJR105W | ADO1 | DELETION | 804.7699461 | 534.7164901 | -11.34420254 | 0.010954812 |
| YDR177W | UBC1 | DAMP-D0333 | 1115.94275 | 845.5237954 | -11.35955614 | 0.010848268 |
| YPL033C | SRL4 | DELETION | 968.2636255 | 697.7260019 | -11.36454109 | 0.010813874 |
| YAL011W | SWC3 | DELETION | 910.8028653 | 639.9239484 | -11.37887788 | 0.010715497 |
| YDR485C | VPS72 | DELETION-41523 | 970.177645 | 698.1361855 | -11.4277131 | 0.010386342 |
| YIR004W | DJP1 | DELETION-41236 | 940.5748991 | 668.345397 | -11.43561225 | 0.010333955 |
| YKL207W | AIM27 | DELETION | 951.314134 | 678.9071586 | -11.44306742 | 0.010284727 |
| YOR322C | LDB19 | DELETION | 807.3656536 | 534.0065648 | -11.48306308 | 0.01002418 |
| YMR283C | RIT1 | DELETION | 969.4106409 | 695.7803055 | -11.4944574 | 0.009951036 |
| YMR077C | VPS20 | DELETION | 665.3325424 | 391.6560715 | -11.49639543 | 0.009938643 |
| YMR137C | PSO2 | DELETION-41717 | 1035.515112 | 761.6861599 | -11.50280072 | 0.00989778 |
| YNR027W | BUD17 | DELETION | 445.0257079 | 171.1233446 | -11.50588454 | 0.00987816 |
| YDL004W | ATP16 | DAMP | 906.3253842 | 631.962099 | -11.52524661 | 0.009755764 |
| YER117W | RPL23B | DELETION-41726 | 860.0248072 | 583.6905392 | -11.60804217 | 0.00924749 |
| YHR118C | ORC6 | DAMP-D1144 | 876.1485736 | 599.0783979 | -11.63895563 | 0.00906386 |
| YBR184W | YBR184W | DELETION | 1054.106788 | 776.9084324 | -11.64434013 | 0.009032211 |
| YLR080W | EMP46 | DELETION | 1091.783176 | 813.0717276 | -11.70790097 | 0.008665995 |
| YOR364W | YOR364W | DELETION-41745 | 1002.234434 | 723.4123604 | -11.71254803 | 0.008639749 |
| YDR071C | PAA1 | DELETION-30646 | 973.0200471 | 446.6550135 | -11.73307064 | 0.008524684 |
| YML003W | YML003W | DELETION | 884.6993037 | 603.7942243 | -11.80004938 | 0.008158626 |
| YGL023C | PIB2 | DELETION-41034 | 865.4126328 | 584.4478185 | -11.80255868 | 0.00814519 |
| YDR477W | SNF1 | DELETION-41620 | 770.4143381 | 488.336769 | -11.84930244 | 0.007898488 |
| YMR235C | RNA1 | DAMP-D0811 | 950.5904212 | 668.4624819 | -11.85141836 | 0.007887481 |
| YOR353C | SOG2 | DAMP-D1010 | 1017.714646 | 735.5328441 | -11.85368098 | 0.007875726 |
| YGR208W | SER2 | DELETION | 645.8546553 | 363.4232212 | -11.86416734 | 0.00782145 |
| YBL016W | FUS3 | DELETION | 1067.696642 | 784.9532254 | -11.87727286 | 0.007754089 |
| YPL203W | TPK2 | DELETION | 1082.666813 | 799.5817919 | -11.89162272 | 0.007680928 |
| YLR282C | YLR282C | DELETION | 1009.18233 | 726.0402917 | -11.89401785 | 0.007668778 |
| YMR139W | RIM11 | DELETION-41725 | 903.2181022 | 619.5896843 | -11.91444934 | 0.007565825 |
| YFR013W | IOC3 | DELETION | 829.453431 | 544.8232133 | -11.95653219 | 0.007357668 |
| YHR118C | ORC6 | DAMP-D1143 | 869.3557178 | 584.5061513 | -11.96574643 | 0.007312783 |
| YLR043C | TRX1 | DELETION | 902.2589519 | 617.303572 | -11.97019135 | 0.007291218 |
| YIL135C | VHS2 | DELETION | 921.6177229 | 635.9415454 | -12.00047008 | 0.007145832 |
| YCR020W-B | HTL1 | DELETION | 715.745577 | 429.48415 | -12.0250548 | 0.007029706 |
| YNL064C | YDJ1 | DELETION | 675.7069087 | 389.0579462 | -12.0413341 | 0.006953747 |
| YLL050C | COF1 | DAMP-D1225 | 981.1877488 | 694.3353042 | -12.04988182 | 0.006914159 |
| YNL107W | YAF9 | DELETION | 1057.885082 | 484.8527315 | -12.0505556 | 0.006911047 |
| YML029W | USA1 | DELETION | 879.0427843 | 592.0189935 | -12.05707961 | 0.00688098 |
| YHR126C | ANS1 | DELETION | 841.9528598 | 553.1398691 | -12.13223897 | 0.006542978 |
| YBR056W | YBR056W | DELETION | 763.1978832 | 472.7636577 | -12.20034259 | 0.006249681 |
| YKR071C | DRE2 | DAMP-D1209 | 969.354847 | 678.2811913 | -12.22720329 | 0.006137295 |
| YAL036C | RBG1 | DELETION | 1199.545114 | 907.486411 | -12.26858243 | 0.005967723 |
| YMR139W | RIM11 | DELETION-31826 | 801.4865401 | 509.2356466 | -12.2766558 | 0.005935134 |
| YJL163C | YJL163C | DELETION-21342 | 867.9274264 | 575.612157 | -12.27936006 | 0.005924255 |
| YMR235C | RNA1 | DAMP-D0611 | 944.3845132 | 651.3652276 | -12.30893384 | 0.00580644 |
| YMR164C | MSS11 | DELETION | 1028.136347 | 735.0449979 | -12.31196104 | 0.0057945 |
| YDR495C | VPS3 | DELETION-41907 | 563.081742 | 268.3120055 | -12.38246547 | 0.00552261 |
| YML021C | UNG1 | DELETION | 904.8990451 | 610.0075089 | -12.38758194 | 0.005503334 |
| YLR417W | VPS36 | DELETION | 681.5047433 | 386.4090738 | -12.39615702 | 0.005471165 |
| YHR108W | GGA2 | DELETION | 872.0878277 | 576.3070243 | -12.42493761 | 0.005364433 |
| YLR455W | YLR455W | DELETION-31048 | 1130.203768 | 834.0083007 | -12.44235649 | 0.005300752 |
| YKR081C | RPF2 | DAMP-D1213 | 876.7963522 | 580.4845142 | -12.44724491 | 0.005283004 |
| YFR019W | FAB1 | DELETION-30644 | 813.6878474 | 516.9649526 | -12.46451227 | 0.005220741 |
| YIL038C | NOT3 | DELETION | 1025.08619 | 728.1947432 | -12.4715927 | 0.005195402 |
| YPL063W | TIM50 | DAMP-D1026 | 948.7594809 | 651.6385237 | -12.48123378 | 0.005161079 |
| YDR477W | SNF1 | DELETION-41320 | 833.8700997 | 534.2518153 | -12.58613963 | 0.004800627 |
| YIL007C | NAS2 | DELETION | 967.3910237 | 667.7512467 | -12.58704247 | 0.004797627 |
| YNL038W | GPI15 | DAMP-D1219 | 800.3676254 | 233.3383083 | -12.61243366 | 0.004713928 |
| YBR209W | YBR209W | DELETION | 910.0042887 | 609.5358391 | -12.62185272 | 0.004683217 |
| YER130C | YER130C | DELETION-42418 | 842.3830938 | 293.4549079 | -12.73426306 | 0.004330431 |
| YGR056W | RSC1 | DELETION | 1211.523287 | 619.2182552 | -12.73936354 | 0.004315011 |
| YCL024W | KCC4 | DELETION | 977.2868474 | 672.9161079 | -12.78577718 | 0.00417696 |
| YLR113W | HOG1 | DELETION | 1094.091169 | 364.876716 | -12.79492199 | 0.004150237 |
| YMR086W | YMR086W | DELETION | 1187.489839 | 880.8567052 | -12.88081415 | 0.003906714 |
| YAL021C | CCR4 | DELETION-40401 | 1380.545557 | 235.8656195 | -12.9165398 | 0.003809302 |
| YOR151C | RPB2 | DAMP-D2509 | 880.2326024 | 572.1661446 | -12.94102414 | 0.003743821 |
| YOR188W | MSB1 | DELETION | 986.5240241 | 678.44812 | -12.94142095 | 0.003742769 |
| YGR057C | LST7 | DELETION | 922.1003725 | 613.4198499 | -12.96681931 | 0.003675943 |
| YDR180W | SCC2 | DAMP-D0337 | 1019.061293 | 710.2766896 | -12.97119145 | 0.00366455 |
| YGR045C | YGR045C | DELETION | 1067.315304 | 757.99019 | -12.99389683 | 0.003605896 |
| YLR447C | VMA6 | DELETION | 323.7653997 | 14.22167185 | -13.00308019 | 0.003582417 |
| YDR070C | FMP16 | DELETION | 1148.37199 | 838.7695744 | -13.0055455 | 0.003576138 |
| YPL238C | YPL238C | DAMP | 1031.271058 | 721.2081957 | -13.0248876 | 0.003527218 |
| YPR070W | MED1 | DELETION | 794.5829989 | 484.4090922 | -13.02955227 | 0.003515511 |
| YJR128W | YJR128W | DELETION | 861.3993886 | 550.5584273 | -13.0575734 | 0.003445931 |
| YML041C | VPS71 | DELETION-41329 | 1024.045165 | 713.0932735 | -13.06223328 | 0.003434482 |
| YHR005C | GPA1 | DELETION-30608 | 901.6770421 | 590.1593543 | -13.08600082 | 0.003376625 |
| YNR052C | POP2 | DELETION-31816 | 559.2708452 | 247.1490592 | -13.11137732 | 0.003315831 |
| YDR099W | BMH2 | DELETION | 1055.017189 | 742.3987197 | -13.13224161 | 0.003266596 |
| YOR151C | RPB2 | DAMP-D2510 | 879.0612066 | 565.0210269 | -13.19196376 | 0.003129325 |
| YAL004W | YAL004W | DELETION | 1101.126082 | 785.5196565 | -13.25775745 | 0.002984201 |
| YKR081C | RPF2 | DAMP-D1013 | 889.3493712 | 573.3155961 | -13.27570922 | 0.002945685 |
| YKR025W | RPC37 | DAMP-D0837 | 794.6886326 | 476.3195605 | -13.37380862 | 0.002743107 |
| YBR023C | CHS3 | DELETION | 1014.873906 | 694.3964657 | -13.46237535 | 0.002571232 |
| YNL062C | GCD10 | DAMP-D1745 | 527.8872631 | 206.0214377 | -13.5206976 | 0.002463489 |
| YDR276C | PMP3 | DELETION | 852.0885105 | 528.887398 | -13.57678934 | 0.00236378 |
| YGR043C | NQM1 | DELETION | 1034.177125 | 710.6173798 | -13.5918545 | 0.002337637 |
| YNL038W | GPI15 | DAMP-D1019 | 781.1005444 | 282.0385625 | -13.60061729 | 0.002322553 |
| YPR069C | SPE3 | DELETION | 935.6423149 | 611.8547085 | -13.60142633 | 0.002321165 |
| YCL059C | KRR1 | DAMP-D1723 | 862.623904 | 538.3586072 | -13.62149279 | 0.002286976 |
| YHR141C | RPL42B | DELETION | 889.0342472 | 258.5718741 | -13.65251897 | 0.002235022 |
| YGL020C | GET1 | DELETION-41108 | 831.5354454 | 305.9202323 | -13.70155785 | 0.002155107 |
| YMR129W | POM152 | DELETION | 930.4179774 | 603.4037048 | -13.7369697 | 0.002099037 |
| YOL121C | RPS19A | DELETION | 851.265645 | 314.9145477 | -13.84472119 | 0.001936554 |
| YMR309C | NIP1 | DAMP-D1203 | 1082.694763 | 412.0054111 | -13.84984422 | 0.001929124 |
| YDR180W | SCC2 | DAMP-D0338 | 1006.446686 | 676.2710321 | -13.86977062 | 0.001900474 |
| YDR182W | CDC1 | DAMP-D0341 | 632.7857575 | 302.0546398 | -13.89310414 | 0.001867422 |
| YGL167C | PMR1 | DELETION-41230 | 915.9858213 | 582.3889316 | -14.01348733 | 0.001705136 |
| YER177W | BMH1 | DELETION-40412 | 899.1080942 | 342.9451001 | -14.04368123 | 0.00166652 |
| YOR008C | SLG1 | DELETION | 891.530981 | 555.9569276 | -14.09654253 | 0.001600845 |
| YMR135W-A | YMR135W-A | DELETION-31814 | 756.939299 | 420.1928807 | -14.14579036 | 0.001541812 |
| YLR248W | RCK2 | DELETION | 893.0540597 | 555.6017976 | -14.17544091 | 0.001507243 |
| YCR079W | PTC6 | DELETION | 900.6555703 | 563.0044203 | -14.18379565 | 0.001497633 |
| YNR031C | SSK2 | DELETION | 860.0615217 | 521.6591701 | -14.21535156 | 0.00146184 |
| YER155C | BEM2 | DELETION-40437 | 799.6849882 | 258.6734632 | -14.25090857 | 0.001422455 |
| YDR454C | GUK1 | DAMP-D1521 | 839.7668054 | 500.2781062 | -14.26098603 | 0.001411472 |
| YDR454C | GUK1 | DAMP-D1522 | 842.0734246 | 502.014978 | -14.28491955 | 0.0013857 |
| YDR182W-A | YDR182W-A | DELETION | 869.162663 | 528.3240141 | -14.3176937 | 0.001351112 |
| YGR071C | YGR071C | DELETION | 1077.372492 | 735.677771 | -14.35365493 | 0.001314078 |
| YMR216C | SKY1 | DELETION | 704.9073667 | 363.0313903 | -14.36126898 | 0.001306358 |
| YJR102C | VPS25 | DELETION | 702.3247393 | 358.9684581 | -14.42345251 | 0.001244858 |
| YJL125C | GCD14 | DAMP | 854.2130451 | 253.6333031 | -14.45157082 | 0.001217936 |
| YLR432W | IMD3 | DELETION | 1278.2037 | 772.884376 | -14.45696887 | 0.00121283 |
| YNL023C | FAP1 | DELETION | 958.7926844 | 614.2675934 | -14.472551 | 0.001198201 |
| YHL015W | RPS20 | DAMP-D1111 | 839.809333 | 495.1838887 | -14.47676656 | 0.001194271 |
| YBR153W | RIB7 | DAMP-D0947 | 654.5711655 | 308.4514796 | -14.53953554 | 0.001137149 |
| YIL006W | YIA6 | DELETION | 870.5648777 | 523.59879 | -14.57509055 | 0.001105924 |
| YLR368W | MDM30 | DELETION | 1373.80379 | 1026.1134 | -14.60551646 | 0.001079835 |
| YBR204C | YBR204C | DELETION | 871.0655038 | 521.9019686 | -14.66739927 | 0.001028521 |
| YPL002C | SNF8 | DELETION | 716.7401081 | 366.1542628 | -14.7271466 | 0.000981127 |
| YBL051C | PIN4 | DELETION | 971.1700541 | 620.1806492 | -14.74409903 | 0.000968053 |
| YJR012C | YJR012C | DAMP | 971.2575684 | 618.124348 | -14.83415482 | 0.000901263 |
| YDR421W | ARO80 | DELETION | 993.3487048 | 636.7458719 | -14.9799037 | 0.000802154 |
| YCL037C | SRO9 | DELETION | 1038.907026 | 680.8945411 | -15.0391193 | 0.000764857 |
| YIL092W | YIL092W | DELETION-21238 | 896.4568541 | 534.6972617 | -15.19652497 | 0.000673397 |
| YDR170C | SEC7 | DAMP-D0325 | 1179.146414 | 815.9663932 | -15.25619324 | 0.000641467 |
| YDL190C | UFD2 | DELETION | 825.4276676 | 461.9031386 | -15.2706651 | 0.000633938 |
| YMR171C | EAR1 | DELETION | 1359.560756 | 756.3727617 | -15.31206992 | 0.000612849 |
| YBR201W | DER1 | DELETION-11023 | 919.909583 | 554.6861943 | -15.34202952 | 0.000597998 |
| YJR129C | YJR129C | DELETION | 825.4667874 | 458.691478 | -15.40722144 | 0.000566833 |
| YBR105C | VID24 | DELETION-32648 | 922.4289707 | 553.7540603 | -15.48701845 | 0.000530745 |
| YGL261C | PAU11 | DELETION | 959.2327028 | 588.6572666 | -15.56685431 | 0.000496791 |
| YNL006W | LST8 | DAMP-D1215 | 877.4573228 | 505.8389688 | -15.61066442 | 0.000479031 |
| YJR075W | HOC1 | DELETION | 903.4237036 | 529.3683349 | -15.7130367 | 0.000439812 |
| YNL320W | YNL320W | DELETION | 999.4910361 | 623.4328321 | -15.7971703 | 0.000409847 |
| YMR309C | NIP1 | DAMP-D1003 | 1077.058497 | 468.0598601 | -15.85840024 | 0.00038925 |
| YDR290W | YDR290W | DELETION | 865.5746528 | 485.9500993 | -15.94698282 | 0.000361158 |
| YDR182W | CDC1 | DAMP-D0342 | 618.5714449 | 238.5249717 | -15.96470651 | 0.00035577 |
| YNL062C | GCD10 | DAMP-D1746 | 566.4227224 | 184.9841241 | -16.02318586 | 0.000338521 |
| YBR178W | YBR178W | DELETION | 991.9596763 | 609.9776039 | -16.04601571 | 0.000332002 |
| YCL059C | KRR1 | DAMP-D1724 | 879.3688759 | 495.4562929 | -16.12711114 | 0.000309783 |
| YGR087C | PDC6 | DELETION | 886.1266277 | 501.7889258 | -16.14496922 | 0.00030508 |
| YIR023W | DAL81 | DELETION-42137 | 827.3818696 | 440.7592937 | -16.24095049 | 0.000280932 |
| YDR293C | SSD1 | DELETION | 1067.510678 | 679.7729909 | -16.28779322 | 0.000269809 |
| YBL084C | CDC27 | DAMP-D0515 | 870.1234092 | 285.081751 | -16.29254899 | 0.000268703 |
| YHR005C | GPA1 | DELETION-40507 | 985.260386 | 591.2736151 | -16.55030005 | 0.000214736 |
| YHL007C | STE20 | DELETION | 873.0497934 | 478.4520921 | -16.57596356 | 0.00020996 |
| YDR531W | CAB1 | DAMP-D1918 | 920.9792799 | 524.5039715 | -16.65483667 | 0.000195898 |
| YLR399C | BDF1 | DELETION-40348 | 824.3248866 | 427.4303871 | -16.67244573 | 0.000192882 |
| YNL006W | LST8 | DAMP-D1015 | 906.3364812 | 507.7757601 | -16.74243912 | 0.000181319 |
| YKR030W | GMH1 | DELETION | 985.9556549 | 584.5023374 | -16.86394913 | 0.000162779 |
| YJR137C | MET5 | DELETION | 1117.665838 | 293.7763168 | -16.91508006 | 0.000155524 |
| YML037C | YML037C | DELETION | 1168.597519 | 765.2513165 | -16.94346404 | 0.000151629 |
| YJL110C | GZF3 | DELETION | 910.2293131 | 501.3312408 | -17.17668279 | 0.000122928 |
| YOL087C | YOL087C | DELETION | 1013.923989 | 603.6544074 | -17.23429615 | 0.000116673 |
| YBR034C | HMT1 | DELETION | 992.8182398 | 580.9235497 | -17.30256246 | 0.000109649 |
| YNR052C | POP2 | DELETION-41715 | 597.8496965 | 183.2687808 | -17.41540341 | 9.89E-05 |
| YBL098W | BNA4 | DELETION | 923.0188203 | 508.3101732 | -17.42076905 | 9.84E-05 |
| YDR127W | ARO1 | DELETION | 842.8332735 | 421.6737736 | -17.69175163 | 7.66E-05 |
| YIR023W | DAL81 | DELETION-32238 | 760.8980109 | 338.5736921 | -17.74068246 | 7.32E-05 |
| YOR328W | PDR10 | DELETION | 939.4333033 | 514.3614633 | -17.85609827 | 6.57E-05 |
| YDR531W | CAB1 | DAMP-D1917 | 925.5617684 | 494.4342448 | -18.11048087 | 5.17E-05 |
| YIR016W | YIR016W | DELETION | 936.0173535 | 504.6681939 | -18.11979119 | 5.13E-05 |
| YLR006C | SSK1 | DELETION-41636 | 837.0973182 | 404.2596816 | -18.18231801 | 4.83E-05 |
| YDR530C | APA2 | DELETION-42715 | 1107.306206 | 351.286688 | -18.29940294 | 4.32E-05 |
| YDR160W | SSY1 | DAMP-D0309 | 687.045538 | 250.573552 | -18.33498703 | 4.17E-05 |
| YML125C | PGA3 | DAMP-D3017 | 620.0128882 | 182.1881179 | -18.39181377 | 3.95E-05 |
| YIR010W | DSN1 | DAMP-D0930 | 772.5548671 | 334.5169189 | -18.40076879 | 3.92E-05 |
| YDR071C | PAA1 | DELETION-40545 | 791.5392846 | 352.4200076 | -18.44619244 | 3.75E-05 |
| YMR135W-A | YMR135W-A | DELETION-41713 | 940.8734551 | 500.5653743 | -18.49613081 | 3.57E-05 |
| YJL182C | YJL182C | DELETION | 969.5182935 | 526.5348116 | -18.60851705 | 3.21E-05 |
| YDR160W | SSY1 | DAMP-D0310 | 697.7644539 | 251.8748083 | -18.73059699 | 2.85E-05 |
| YKR025W | RPC37 | DAMP-D0637 | 781.8012139 | 332.5558534 | -18.87156134 | 2.48E-05 |
| YER130C | YER130C | DELETION-42118 | 846.315479 | 394.2685276 | -18.98924847 | 2.21E-05 |
| YMR173W | DDR48 | DELETION-41733 | 1029.013203 | 576.6781731 | -19.00134985 | 2.18E-05 |
| YDR094W | YDR094W | DELETION | 871.980019 | 419.4095867 | -19.01123845 | 2.16E-05 |
| YCL045C | EMC1 | DELETION | 923.0486172 | 469.8087804 | -19.0393583 | 2.10E-05 |
| YHR198C | AIM18 | DELETION | 1172.498247 | 715.6192887 | -19.19222777 | 1.80E-05 |
| YIR010W | DSN1 | DAMP-D0929 | 797.7334846 | 336.9301067 | -19.35708185 | 1.53E-05 |
| YBL094C | YBL094C | DELETION | 1224.087297 | 268.7672038 | -19.54347758 | 1.26E-05 |
| YBR162C | TOS1 | DELETION-41101 | 1517.150731 | 1049.513507 | -19.64415293 | 1.14E-05 |
| YJL204C | RCY1 | DELETION | 738.7046443 | 262.0293757 | -20.0238163 | 7.72E-06 |
| YJL128C | PBS2 | DELETION-31044 | 737.0934222 | 260.2980276 | -20.02886246 | 7.68E-06 |
| YLR399C | BDF1 | DELETION-40248 | 934.1433059 | 441.5136404 | -20.69401661 | 3.80E-06 |
| YNR010W | CSE2 | DELETION | 743.1641123 | 245.9271078 | -20.88755824 | 3.09E-06 |
| YGR212W | SLI1 | DELETION | 868.3521063 | 362.9877743 | -21.2289649 | 2.13E-06 |
| YGR213C | RTA1 | DELETION | 839.258855 | 333.1533242 | -21.26010063 | 2.06E-06 |
| YBR276C | PPS1 | DELETION | 1163.09477 | 654.7635944 | -21.35359385 | 1.86E-06 |
| YDR530C | APA2 | DELETION-32816 | 940.2077418 | 389.8869702 | -23.11746122 | 2.46E-07 |
| YDL100C | GET3 | DELETION | 919.2919812 | 360.4844148 | -23.47396811 | 1.61E-07 |
| YDL173W | PAR32 | DELETION | 904.3669005 | 330.8185206 | -24.09318912 | 7.57E-08 |
| YML125C | PGA3 | DAMP-D3217 | 730.7872092 | 144.8616757 | -24.61311929 | 3.96E-08 |
| YBR125C | PTC4 | DELETION | 829.1215189 | 234.4723711 | -24.97957432 | 2.49E-08 |
| YJL195C | YJL195C | DAMP | 933.8067536 | 332.538439 | -25.2576273 | 1.74E-08 |
| YBL084C | CDC27 | DAMP-D0516 | 901.3485691 | 289.6545425 | -25.69558277 | 9.85E-09 |
| YNL291C | MID1 | DELETION | 1069.734746 | 412.524316 | -27.60760162 | 7.36E-10 |
| YIL105C | SLM1 | DELETION | 939.4504771 | 214.6960752 | -30.44493801 | 1.13E-11 |
| YMR145C | NDE1 | DELETION | 1210.648644 | 181.5748604 | -43.22855779 | 6.01E-22 |

**Supplementary Table 3: List of all hits identified in the chemical genomic screen**
