# Supplementary material for: The GARP complex is required for cellular sphingolipid homeostasis
Source: eLife. 2015 Sep 10;4:e08712. doi: 10.7554/eLife.08712 (PMC4600884; doi:10.7554/eLife.08712)
Supplement: Supplementary file 4. — Phenotypes of different trafficking complexes identified in the chemical genetic screen. DOI: http://dx.doi.org/10.7554/eLife.08712.021 [file elife08712s004.docx]

| **Supplementary Table 4** | | | | |
| --- | --- | --- | --- | --- |
|  |  |  |  |  |
|  |  |  |  |  |
| ORF | Gene Name | Mutation | T-score | P value |
| **GARP complex** | | | | |
| YDR027C | VPS54 | DELETION | 46.84 | 1.12E-23 |
| YDR484W | VPS52 | DELETION-31620 | 30.31 | 8.99E-11 |
| YDR484W | VPS52 | DELETION-41519 | 29.77 | 1.94E-10 |
| YJL029C | VPS53 | DELETION | 28.51 | 1.08E-09 |
| YKR020W | VPS51 | DELETION | 12.53 | 7.64E-03 |
|  |  |  |  |  |
| **RETROMER** | | | | |
| YHR012W | VPS29 | DELETION | 16.39 | 4.73E-04 |
| YJL053W | PEP8 | DELETION | 15.76 | 7.81E-04 |
| YOR132W | VPS17 | DELETION | 11.85 | 1.17E-02 |
| YJL154C | VPS35 | DELETION | 10.87 | 2.09E-02 |
| YOR069W | VPS5 | DELETION-12532 | 7.77 | 1.00E-01 |
| YOR069W | VPS5 | DELETION-43203 | 7.13 | 1.32E-01 |
|  |  |  |  |  |
| **COG complex** | | | | |
| YGL223C | COG1 | DELETION-40344 | 10.46 | 2.62E-02 |
| YGL223C | COG1 | DELETION-40244 | 9.04 | 5.53E-02 |
| YNL041C | COG6 | DELETION | -6.00 | 1.75E-01 |
| YGR120C | COG2 | DAMP-D0728 | -5.53 | 2.11E-01 |
| YGR120C | COG2 | DAMP-D0727 | -5.29 | 2.30E-01 |
| YPR105C | COG4 | DAMP-D1834 | 2.49 | 6.09E-01 |
| YNL051W | COG5 | DELETION | -2.13 | 6.20E-01 |
| YPR105C | COG4 | DAMP-D1048 | -1.91 | 6.54E-01 |
| YGL005C | COG7 | DELETION | 1.20 | 8.14E-01 |
| YML071C | COG8 | DELETION | 0.34 | 9.59E-01 |
|  |  |  |  |  |
| **TRAPP I complex** | | | | |
| YKR068C | BET3 | DAMP-D1205 | 8.26 | 8.02E-02 |
| YML077W | BET5 | DAMP-D3013 | 3.29 | 4.94E-01 |
| YDR472W | TRS31 | DAMP-D1538 | 3.12 | 5.17E-01 |
| YBR254C | TRS20 | DAMP-D1344 | 2.80 | 5.63E-01 |
| YBR254C | TRS20 | DAMP-D1343 | 2.43 | 6.18E-01 |
| YOR115C | TRS33 | DELETION | 2.30 | 6.38E-01 |
| YDR472W | TRS31 | DAMP-D1537 | 1.71 | 7.30E-01 |
| YDR246W | TRS23 | DAMP | 1.14 | 8.25E-01 |
| YML077W | BET5 | DAMP-D3213 | 1.02 | 8.45E-01 |
|  |  |  |  |  |
| **TRAPP II complex** | | | | |
| YDR407C | TRS120 | DAMP-D1510 | 14.86 | 1.55E-03 |
| YDR407C | TRS120 | DAMP-D1509 | 14.23 | 2.43E-03 |
| YKR068C | BET3 | DAMP-D1205 | 8.26 | 8.02E-02 |
| YEL048C | TCA17 | DELETION | 4.91 | 3.03E-01 |
| YMR218C | TRS130 | DAMP | -3.09 | 4.78E-01 |
| YML077W | BET5 | DAMP-D3013 | 3.29 | 4.94E-01 |
| YDR472W | TRS31 | DAMP-D1538 | 3.12 | 5.17E-01 |
| YBR254C | TRS20 | DAMP-D1344 | 2.80 | 5.63E-01 |
| YBR254C | TRS20 | DAMP-D1343 | 2.43 | 6.18E-01 |
| YOR115C | TRS33 | DELETION | 2.30 | 6.38E-01 |
| YDR472W | TRS31 | DAMP-D1537 | 1.71 | 7.30E-01 |
| YDR246W | TRS23 | DAMP | 1.14 | 8.25E-01 |
| YML077W | BET5 | DAMP-D3213 | 1.02 | 8.45E-01 |
|  |  |  |  |  |
| **TRAPP III complex** | | | | |
| YKR068C | BET3 | DAMP-D1205 | 8.26 | 8.02E-02 |
| YDR108W | GSG1 | DELETION | 3.71 | 4.40E-01 |
| YML077W | BET5 | DAMP-D3013 | 3.29 | 4.94E-01 |
| YDR472W | TRS31 | DAMP-D1538 | 3.12 | 5.17E-01 |
| YBR254C | TRS20 | DAMP-D1344 | 2.80 | 5.63E-01 |
| YBR254C | TRS20 | DAMP-D1343 | 2.43 | 6.18E-01 |
| YOR115C | TRS33 | DELETION | 2.30 | 6.38E-01 |
| YDR472W | TRS31 | DAMP-D1537 | 1.71 | 7.30E-01 |
| YDR246W | TRS23 | DAMP | 1.14 | 8.25E-01 |
| YML077W | BET5 | DAMP-D3213 | 1.02 | 8.45E-01 |

**Supplementary Table 4: Phenotypes of different trafficking complexes identified in the chemical genetic screen**
